# Genetic Variation and Quantitative Trait Loci Analysis of the Maize Ionome in Response to Phosphorus Fertilisation

**DOI:** 10.1111/pce.70174

**Published:** 2025-09-03

**Authors:** Sandra Roller, Thea M. Weiß, Volker Hahn, Tobias Würschum

**Affiliations:** ^1^ Institute of Plant Breeding, Seed Science and Population Genetics University of Hohenheim Stuttgart Germany; ^2^ State Plant Breeding Institute University of Hohenheim Stuttgart Germany

**Keywords:** analysis of variance, environment, genome‐wide association study, ionome, landraces, minerals, phosphorus, *Zea mays*

## Abstract

Improving the nutritional quality of crops is crucial for human health, livestock, and agricultural productivity, especially on nutrient‐limited soils. To address this, we investigated the variation and the genetic basis of mineral content, including, among others, calcium, iron, phosphorus, and zinc, in a diverse panel of maize (*Zea mays* L.) grown across environments. Our results show that genetic variation significantly contributes to differences in mineral content. Genotype‐by‐environment interaction and environmental factors, such as reduced phosphorus fertilisation, substantially impact the ionome composition, particularly decreasing zinc content and altering grain quality. Correlations between the 12 minerals were mostly positive, with variation observed in mineral composition between tissues and in translocation from vegetative to generative tissue. In addition, elite lines exhibited distinct mineral profiles compared to landraces. Genome‐wide association mapping revealed a quantitative inheritance of the minerals and few common quantitative trait loci. Significantly associated markers were found in proximity to candidate genes involved in processes like mineral transport, detoxification and storage, which represent potential targets for marker‐assisted selection to improve nutritional quality in maize. In conclusion, our results highlight the temporal and spatial dynamics of the maize ionome as a basis toward its targeted design for future agriculture.

## Summary statement

- In maize (*Zea mays* L.), significant genetic variation exists for mineral content, with genotype‐by‐environment interactions influencing the ionome.
- Phosphorus fertilisation alters mineral profiles, and genome‐wide association mapping revealed a quantitative inheritance and common QTL between minerals.

## 1 Introduction

Advances in plant nutrition and biofortification have encouraged extensive research into genetic and environmental factors that govern nutrient uptake and utilisation. Understanding the plant's strategies for these processes is essential in a world of nutrient deficiency in some regions while others suffer from over‐fertilisation (Penuelas et al. 2023), and will support further advancements in fertiliser‐use efficiency as well as in determining the optimum fertiliser composition. In addition, understanding the relationships between the mineral contents in a plant and their interaction with the environment is crucial for a targeted design of the crops' mineral composition.

This complex topic can be addressed by investigating the ionome. The ionome refers to the full spectrum of inorganic minerals in a biological system, representing its elemental composition (Lahner et al. 2003). Of particular interest are the associations between the minerals and their response to environmental changes. Research in ionomics has the potential to close the knowledge gap of traditional single‐element research compared with the reality of complex mineral–mineral interactions (Baxter et al. 2012; Akcura and Kokten 2017; Naciri et al. 2022). Elements may share chemical properties and are known to compete with each other for uptake (Ozaki et al. 2005; Arredondo et al. 2006; Rizwan et al. 2019) or have been shown to be taken up by mineral unspecific transporters, for example iron (Fe) (Korshunova et al. 1999; Vert et al. 2021). Additionally, ionomic screening of soybean [*Glycine max* (L.) Merr.] (Ziegler et al. 2013) and Arabidopsis (Lahner et al. 2003) identified mutants with multi‐element phenotypes. Therefore, a restricted view of a single element is unlikely to provide the complete picture. The ionome has also been identified as a potential indicator for other biological functions. In the study of Baxter et al. (2009), for example, a decrease in the accumulation of Ca, Mn, and Zn and an increase in the accumulation of Na, S, K, As, Se, and Mo were associated with changes in suberin and water transport and therefore with changed drought tolerance in Arabidopsis. In another case, the leaf ionome of Arabidopsis could predict the plant's Fe status better than the Fe concentration itself (Baxter et al. 2008). Consequently, the ionomic profile could be indicative of the nutritional and physiological status of the plant.

The complex nature of the ionome poses several challenges to scientists and plant breeders, but it also offers the opportunity to face agriculture on nutrient‐deficient land and level the path for future biofortification approaches. Biofortification is the process of increasing the nutritional value of crops through breeding or fertiliser application, aiming to enhance the content of essential vitamins and minerals in the edible parts of the plants (Bouis et al. 2014). The challenge lies in the disproportionate allocation of minerals between the different plant organs, making it essential for any approach to focus on enhancing nutrient levels specifically in the edible parts. While it is possible to increase certain mineral levels by providing them to the plant via external fertilisation, extensive application can have negative environmental consequences, such as for phosphorus (P) (Isermann 1990; Sharpley 1995), which like potassium (K) is a finite minable fertiliser with low efficiency (Scholz et al. 2013; Dhillon et al. 2019). Another method would be to increase the nutritional value of crops by conventional breeding. Genetic variation in uptake efficiency and allocation to edible tissue in plants offers an opportunity to enhance crop nutrient levels through breeding. For example, Zinc (Zn) uptake efficiency varies not only between crops but also between genotypes within one crop (Graham and Rengel 1993). Past efforts for mineral biofortification, also in maize (*Zea mays* L.), often focused on Fe and Zn, as scarcity can lead to Fe deficiency‐induced anaemia or slowed growth due to Zn deficiency (Lung'aho et al. 2011; Obaid et al. 2022).

To advance biofortification by plant breeding, understanding the network of the ionome and its genetic architecture will be of utmost importance. However, dissecting the genetic architecture of the ionome will be a complicated endeavour, as it was shown to be governed by many quantitative trait loci (QTL), each contributing a small amount of the genotypic variation. In their study about seed iron and zinc (Zn) content in common bean (*Phaseolus vulgaris* L.), Blair et al. (2009) demonstrated a quantitative inheritance based on the large number of identified QTL. Consistent results were also shown in Arabidopsis (Vreugdenhil et al. 2004), Chinese cabbage (*Brassica rapa* L. ssp. *pekinensis*) (Wu et al. 2008) and rice (*Oryza sativa* ssp. *indica*) (Garcia‐Oliveira et al. 2009). A large number of QTL for mineral concentrations have been reported this way, even leading to the identification of functional Zn and Fe (Li et al. 2013), Mg (Li et al. 2018) and P transporters (Nagy et al. 2006) in maize. Although ionomics research has been conducted in various crops (Baxter et al. 2008; Stich et al. 2020; Shariatipour et al. 2021b), the unique or joint genetic basis of the minerals remains a complex field of research.

The nutrient composition of plants, and therefore the understanding of its genetic and environmental regulation and effect on growth, has until now often focused on model plants grown under controlled conditions in greenhouses or climate chambers (Lahner et al. 2003; Baxter et al. 2008; Du et al. 2020). While this is an efficient way to identify underlying genes, these results must be confirmed in crops grown under field conditions. Under field conditions, P is a growth‐limiting mineral in some regions of the world, while other regions like Central Europe mainly seek to reduce P fertilisation while maintaining yield. This is why understanding the interaction of the plant ionome in different P soil levels is imperative and can give further directions for plant breeding efforts in improving nutrient efficiency. Given its worldwide acreage (FAOSTAT 2024) and relevance in human and animal nutrition (Ranum et al. 2014), maize is an excellent candidate for this field of research.

By analyzing the ionome profile of maize, genetic factors that influence mineral content can be identified, which is key for developing crop varieties with improved nutritional value and fertiliser use efficiency. To this end, we performed an extensive study with maize evaluated at three environments, where we characterised 12 minerals, including calcium (Ca), copper (Cu), Fe, K, magnesium (Mg) and P. The objectives of this study were to (i) compare the mineral composition in early and late vegetative tissue as well as in grains, and the translocation from vegetative to generative plant organs, (ii) examine the network of relationships between minerals in the different tissues, (iii) assess the impact of two P fertilisation regimes on the ionome, (iv) analyse and compare the ionome in a diverse panel of elite and landrace maize lines, and (v) determine the genetic architecture underlying mineral concentrations and contents within each group, tissue and P fertilisation treatment.

## 2 Materials and Methods

### 2.1 Field Trials

The plant material consisted of 198 doubled haploid lines from six European landraces (LR), 100 Flint elite lines (Flint) and 100 Dent elite lines (Dent), which were previously described by Böhm et al. (2017) and Würschum et al. (2022). The landraces originate from France (‘Campan Galade’, CAMP, *n* = 10), Germany (‘Gelber Badischer Landmais’, GELB, *n* = 32; ‘Strenzfelder’, STRE, *n* = 30), Romania (‘Satu Mare’, SATU, *n* = 53) and Switzerland (‘St Galler Rheintaler’, STGA, *n* = 14). The Dent and Flint lines are from the breeding programme of the University of Hohenheim in Germany.

The field trials were conducted in 2019 and 2020 and were detailed in Weiß et al. (2022). Three environments (location × year combinations) are mentioned in the following, that is Hohenheim (HOH) in the years 2019 (HOH19) and 2020 (HOH20) and Eckartsweier in 2020 (EWE20). In the field trials, a treatment of 115 kg triple superphosphate (TSP) starter fertiliser per ha, resulting in 52.9 kg P/ha, was realised during sowing, next to a control (CO) without starter fertiliser. The experimental design for both treatments was an alpha lattice design with two replications. The block size was 5, with 90 incomplete blocks per replication in HOH19 and 80 in HOH20 and EWE20, due to a reduction in the number of genotypes. The plots comprised two rows with a spacing of 0.75 m, a length of 4 m, and a density of 8.66 plants per m^2^.

### 2.2 Mineral Traits

Tissue samples were taken from early (six‐leaf stage) biomass tissue (BM_early_) and of plants at maturity right before harvest, separated into biomass (BM_mature_) and grain (Grain). Mature plants were identified as harvest‐ready based on fully dried leaves and hardened cobs. To collect samples, four individuals for BM_early_ and three individuals for BM_mature_ were chosen randomly from a plot and pooled together. For BM_mature_ the ears were removed from the plants. During harvest, grain samples were taken from the whole plot. After drying, samples were milled to 1 mm and the mineral concentration was measured through X‐ray fluorescence (S2 Puma, Bruker) in parts per million [ppm]. The following 12 minerals were assessed in this study: calcium (Ca), chromium (Cr), copper (Cu), iron (Fe), potassium (K), magnesium (Mg), manganese (Mn), nickel (Ni), P, sulphur (S), strontium (Sr) and zinc (Zn). In 2019, no data was collected from late biomass samples at the Hohenheim location due to harvest logistics. Additionally, no data are available for Cr in grain samples across all environments, and there are also missing data for Cu and Ni in grain samples, with the latter also lacking data in early biomass at Hohenheim in 2019.

The average mineral content per plant [mg/plant] was calculated by multiplying the concentration [ppm] with the sample dry weight of the biomass or the grains. The mineral content represents the total uptake and translocation of mineral elements into the respective tissue. This approach accounts for variations in biomass or grain yield, which in our case was primarily influenced by the different subpopulations (Stich et al. 2020). The Harvest Index (HI) for each mineral was calculated by dividing the grain mineral content by the mineral content in the biomass at maturity.

### 2.3 Statistical Analysis

For all following analyses, the best linear unbiased estimates (BLUEs) were calculated in RStudio version 3.5.3 (RStudio 2020) with ASRemL‐R 4.0 (Butler et al. 2009). BLUEs were used to obtain unbiased genotype means by correcting for environmental and design effects, ensuring reliable comparisons between treatments and tissues. The raw data underwent strict outlier control by visual means and the Bonferroni‐Holm method (Bernal‐Vasquez et al. 2016). To calculate the BLUEs in the multi‐environment trial, the following mixed model was applied separately for each treatment and tissue:

$$y_{ijkh}=\mu +G_{i}+E_{h}+{\left(GE\right)}_{ih}+R_{jh}+B_{hjk}+\varepsilon_{ijk}$$

*μ*: the overall mean

*G*_*i*_: the effect of *i*‐th genotype

*E*_*h:*_ the effect of the *h*‐th environment

*(GE)*_*ih*_ *:* the interaction effect of *i*‐th genotype and *h*‐th environment

*R*_*hj*_: the effect of the *j*‐th replicate nested within the *h*‐th environment

*B*_*hjk*_: the effect of the *k*‐th block nested within the *h*‐th environment and *j*‐th replicate

*ε*_*ijk*_: the residual error effect

Additionally, to enable a Genotype‐by‐Environment analysis (GGE), BLUEs were also calculated separately within each environment using the following model:

$$y_{ijk}=\mu +G_{i}+R_{j}+B_{jk}+\varepsilon_{ijk}$$

*μ*: the overall mean

*G*_*i*_: the effect of *i*‐th genotype

*R*_*j*_: the effect of *j*‐th replicate

*B*_*jk*_: the effect of the *k*‐th block nested within the *j*‐th replicate

*ε*_*ijk*_: the residual error effect

Genotype plus genotype‐versus‐environment interaction (GGE) analysis and biplot visualisation were conducted using the R package metan (Olivoto and Lúcio 2020). This method facilitates the exploration of genotype performance across diverse environments by summarising both genotype main effects and genotype‐by‐environment interactions. This data analysis has been used in several studies for various crops (Hassani et al. 2018; Shahriari et al. 2018; Döttinger et al. 2023). Environments were defined as the combination of location, year, and treatment (Location × Year × Treatment). The GGE analysis was exemplarily performed for the selected traits Fe, P and Zn.

To assess the variance components within each treatment, all factors, apart from the mean, were regarded as random in the model. Heritability (*h* ^*2*^) was determined following Piepho and Möhring (2007) as:

$$H^{2}=1-\frac{A_{\mathrm{tt}}}{\left(2\sigma_{g}^{2}\right)},$$

where *H* ^*2*^ denotes the generalised heritability, *A*_*tt*_ the average pairwise prediction error variance for the genotypic term, and $\sigma_{g}^{2}$ the genotypic variance estimate.

To estimate the genotype‐by‐treatment interaction variance and the variance components across P levels, an expanded model was employed across the two treatments. This model included the treatment effect as a fixed term and its interaction effects as random terms.

$$y_{ijkhp}=\mu +G_{i}+E_{h}+T_{i}+{\left(GE\right)}_{ih}+{\left(GT\right)}_{ip}+{\left(ET\right)}_{hp}+{\left(GET\right)}_{ihp}+R_{jh}+B_{hjk}+\varepsilon_{ijk}$$

*T*_*p*_: the effect of the p‐th treatment

*(GE)*_*ih*_: the interaction effect of *i*‐th genotype and *h*‐th environment

*(GT)*_*ip*_ *:* the interaction effect of *i*‐th genotype and *p*‐th treatment

*(ET)*_*hp*_ *:* the interaction effect of *i*‐th environment and *p*‐th treatment

*(GET)*_*ihp*_: the interaction effect of *i*‐th genotype and *h*‐th environment and *p*‐th treatment

### 2.4 Genome‐Wide Association Study

The characteristics of the genotypic data was previously described in Weiß et al. (2022) and Würschum et al. (2022). Genome‐wide association mapping was conducted with the GAPIT R package version 3 (Wang and Zhang 2021) using the Bayesian‐information and Linkage‐disequilibrium Iteratively Nested Keyway (BLINK) model (Huang et al. 2019). Marker data were obtained by the MaizeSNP50 BeadChip from Illumina® (Ganal et al. 2011).

To detect QTL linked to the difference in the concentration and content of mineral elements between the two examined P regimes (TSP, CO) and between the tissues, the analysis was performed separately in these subsets. Likewise, the association mapping was done in each of the subpopulations (Dent, Flint, LR) and in addition in the whole diversity panel across all genotypes (All).

In the first step, markers with more than 5% heterozygosity or more than 50% missing values were removed. In addition, genotypes with more than 20% missing values were removed. Following this, further quality control was conducted for each of the subpopulations (All, Dent, Flint, LR). Heterozygous markers were removed and the minor allele frequency was filtered for 3%. For each subpopulation, imputation was then performed separately with BEAGLE 5.0 (Browning et al. 2018) and imputed data was filtered for a minor allele frequency greater than 5%.

A Bonferroni‐corrected threshold of *p* < 0.05 was used to take multiple testing into account. To calculate the total proportion of the explained genotypic variance (*p*_*G*_), significant QTL were fitted in a linear model in the order of the strength of their association. The formula used was:

$$p_{G}=R_{\mathrm{adj}}^{2}/h^{²}$$

To estimate the *p*_*G*_ of each QTL, the sum of squares for the specific SNP was divided by the total sum of squares (Neuweiler et al. 2022).

The B73 reference genome V4 was used to annotate candidate genes through the maizeGDB genome browser (Woodhouse et al. 2021). Candidate genes were considered if they were within 50 kbp of the significant SNP.

## 3 Results

### 3.1 Mineral Traits Show Large Genetic Variation and High Heritability

**Figure 1:**
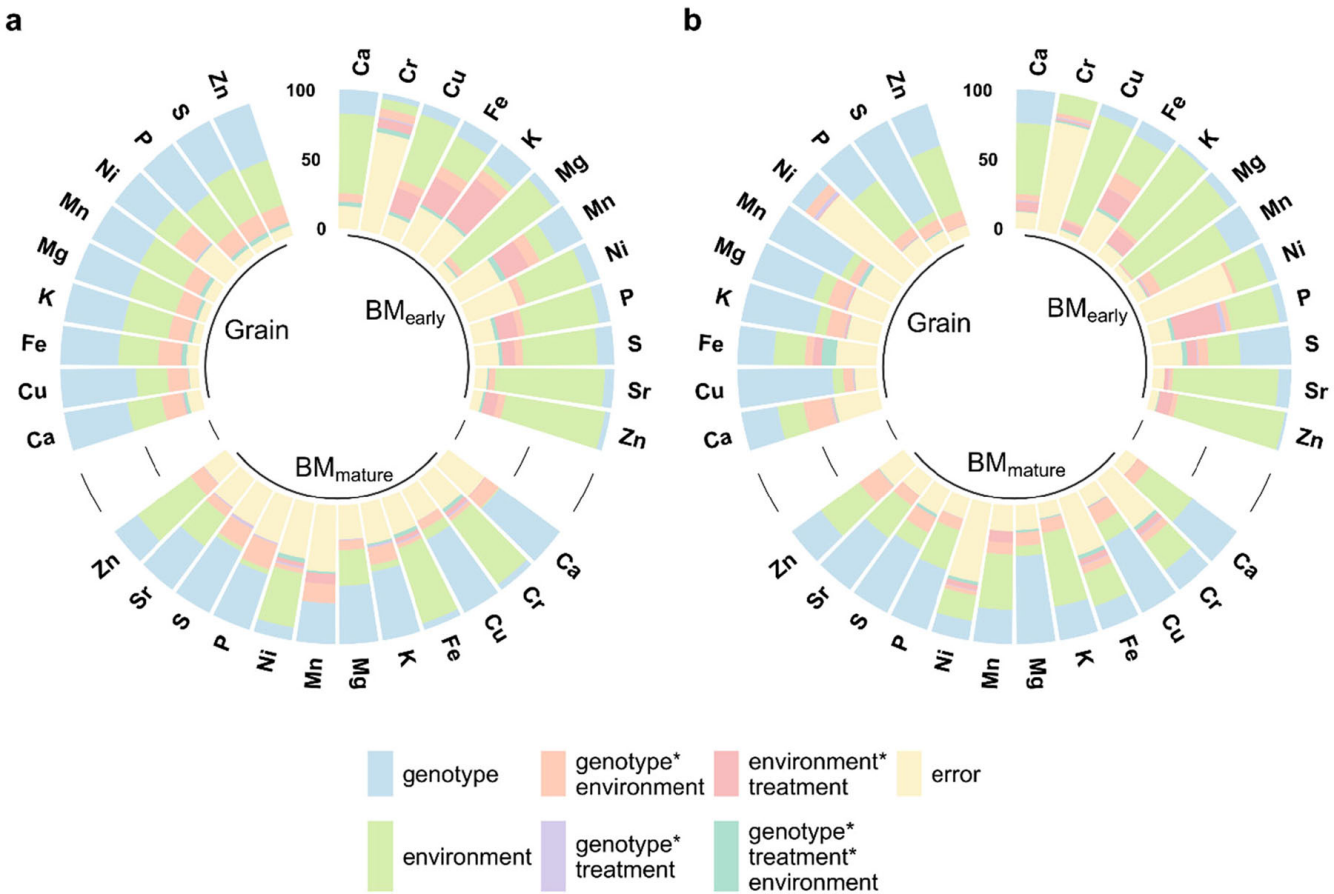
Analysis of the phenotypic variation. Shown are percentage contributions of variance components from the analysis across treatments for (a) mineral content and (b) mineral concentration in early (BM_early_) and late (BM_mature_) biomass and in grain (Grain) tissue. [Color figure can be viewed at wileyonlinelibrary.com]

The ionome of 398 diverse maize lines was evaluated in three tissues from early and late plant developmental stages as well as under two P fertiliser treatments (CO, TSP) at three environments. Most minerals showed significant genotypic variance in all tissue types in both treatments. The genotype‐by‐treatment interaction variance was comparably small and mostly nonsignificant. In contrast, the genotype‐by‐environment and the treatment‐by‐environment interaction variance were significant for almost all minerals. The three‐way interaction variance genotype‐by‐environment‐by‐treatment was for most minerals, like for Cu and Sr, significant in early biomass tissue and for some minerals, like S, Fe and Mg, also in all three tissues. Taken together, all minerals were affected by the combined effect of these three factors in at least one of the studied plant tissues. For minerals such as Fe, K, Mg, P and S, the largest proportion of variance in early biomass was attributable to the environment, which had a distinctively larger effect in this tissue than in the biomass or grain at maturity (Figure 1).

The heritability strongly depended on the trait and ranged from zero for Cr (BM_early_, TSP) to 0.94 for S (Grain, CO). Heritability of mineral content was generally higher in grain samples than in early and late biomass, which was less pronounced for mineral concentration. A difference between the two P treatments was not evident (Supporting Information S1: Figure S1).

### 3.2 Ionome Variation Between Genetic Material and Treatment Conditions

We further evaluated the variation of the ionome in different genetic material and under the two P fertiliser treatments. The effect of the P treatment was not consistent across all three tissue types, as there was interaction between the treatment and the tissue type. For minerals such as P, Mn, Ca, Cu, and K, the added P starter fertiliser influenced the mineral content, at least in the vegetative tissue. For example, we observed a 36.50% increase in Mn content in early plant tissue under fertilised conditions and an increase of 31.15% in Fe. In contrast, Zn content decreased by 10.27% and Zn concentration by 14.60% with the addition of starter fertiliser. For the grains, only Ni and Zn content and concentration showed a significant response to the addition of starter fertilisation, both increasing by 18.23 and 8.70% in content, respectively.

**Figure 2:**
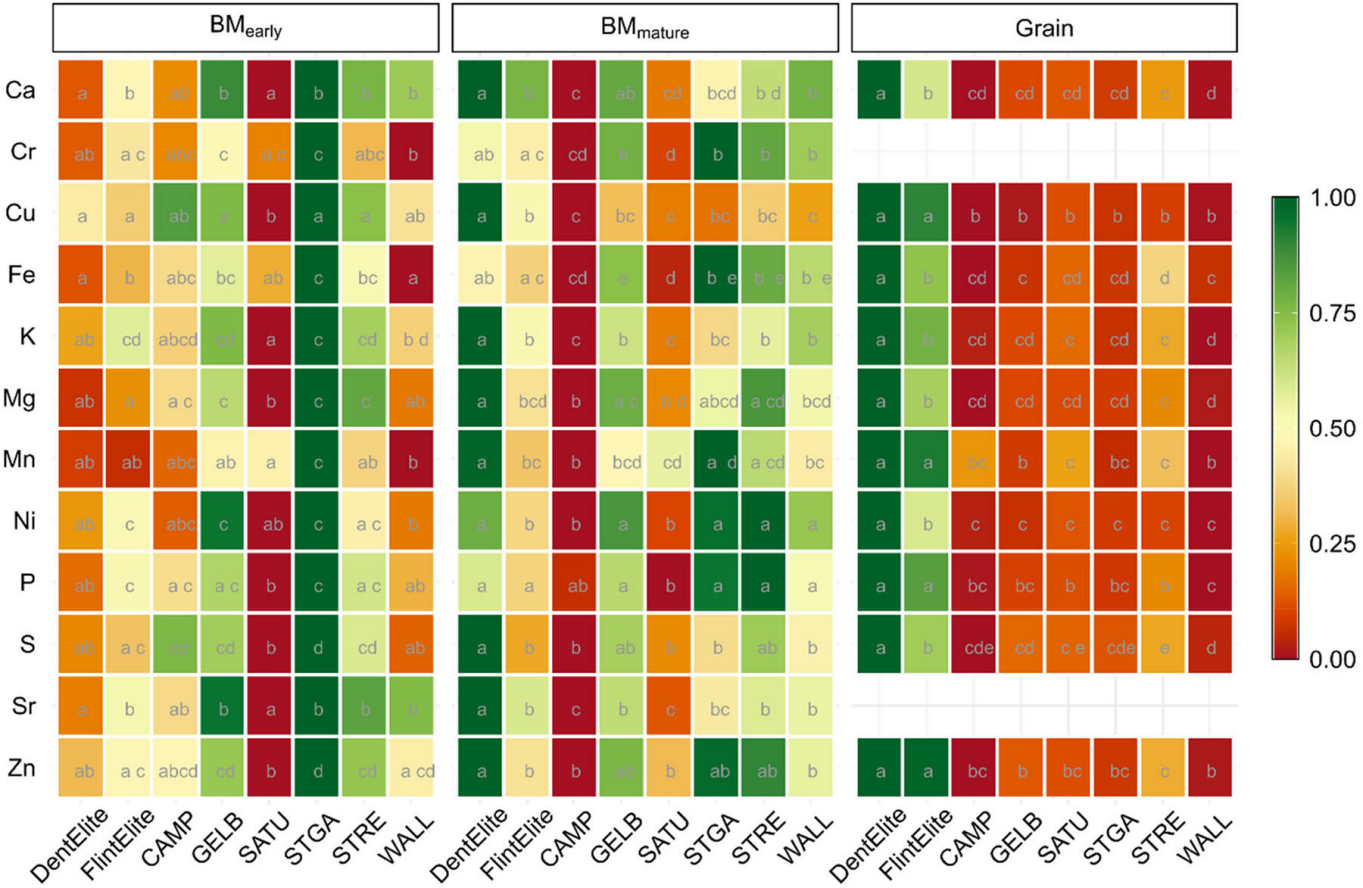
Ionome variation between genetic groups and tissues. Displayed is the standardised mean mineral content for the Dent and Flint elite lines and for each of the landraces in the control treatment, separated by tissue (BM_early_, BM_mature_, Grain). The colour indicates the mean standardised value. Letters within the panels denote statistically significant differences by analysis of variance based on a linear model fit among genetic groups for a given mineral. [Color figure can be viewed at wileyonlinelibrary.com]

Another significant factor responsible for differences in mineral uptake was the subpopulation. During early development, the landrace ‘St Galler Rheintaler’ (STGA) stood out with a high mineral content in their biomass, whereas the landrace ‘Campan Galade’ (CAMP) showed low values in mature biomass and the landrace ‘Satu Mare’ (SATU) had consistently lower mineral content in biomass compared to the other groups (Figure 2). The Dent lines had considerably lower values than the Flint lines. While the elite Dent, Flint or landrace lines showed differences in mineral accumulation, the contrast in mineral content was most pronounced for K, P, Mg, Zn, Ni and S in grains of elite Dent and landrace lines, as the grain content of the landraces was significantly lower than that of elite lines. When examining concentrations [ppm] without accounting for biomass or grain mass, for some minerals the reverse is apparent (Supporting Information S1: Figure S2). Especially for Dent lines, the significantly higher grain yield leads to lower mineral concentration values, which can likely be attributed to a dilution effect (Stich et al. 2020).

The differences in mineral content among subpopulations were further supported by the GGE biplot analysis (Supporting Information S1: Figure S2d–f). While landraces showed the highest mineral content in early biomass – as indicated by the arrow at the extremes of the biplot vector – they also exhibited high instability across environments (reflected in their distance from the origin). In contrast, the biplot for grain tissue (Supporting Information S1: Figure S2f) showed that high‐performing and more stable genotypes were predominantly Dent and Flint lines. For example, the landrace line SATU.135 had high Fe content in grain but showed considerable instability, whereas Dent lines such as P353 and P223 combined high Fe content with greater stability. The which‐won‐where biplot (Supporting Information S1: Figure S3a–c) details the distribution of the genotypes across the environments. The genotypes at the edges of the polygon are the most responsive to the respective environment of their sector. Notably, these positions were mainly occupied with landraces. Genotypes near the centre of the biplot tend to perform similarly across environments and show little responsiveness to environmental variation. In contrast, genotypes on the edges are those with the highest values in at least one group of environments within the same sector (Yan et al. 2007). As an example, S066, a Flint line, was the highest‐yielding in three of the environments for Zn content in grain.

Nevertheless, the principal component analysis based on the ionome data showed a considerable overlap of the subpopulations over all tissues, irrespective of the treatment. Only for grain did a slight separation of Dent and landraces become evident (Supporting Information S1: Figure S4).

**Figure 3:**
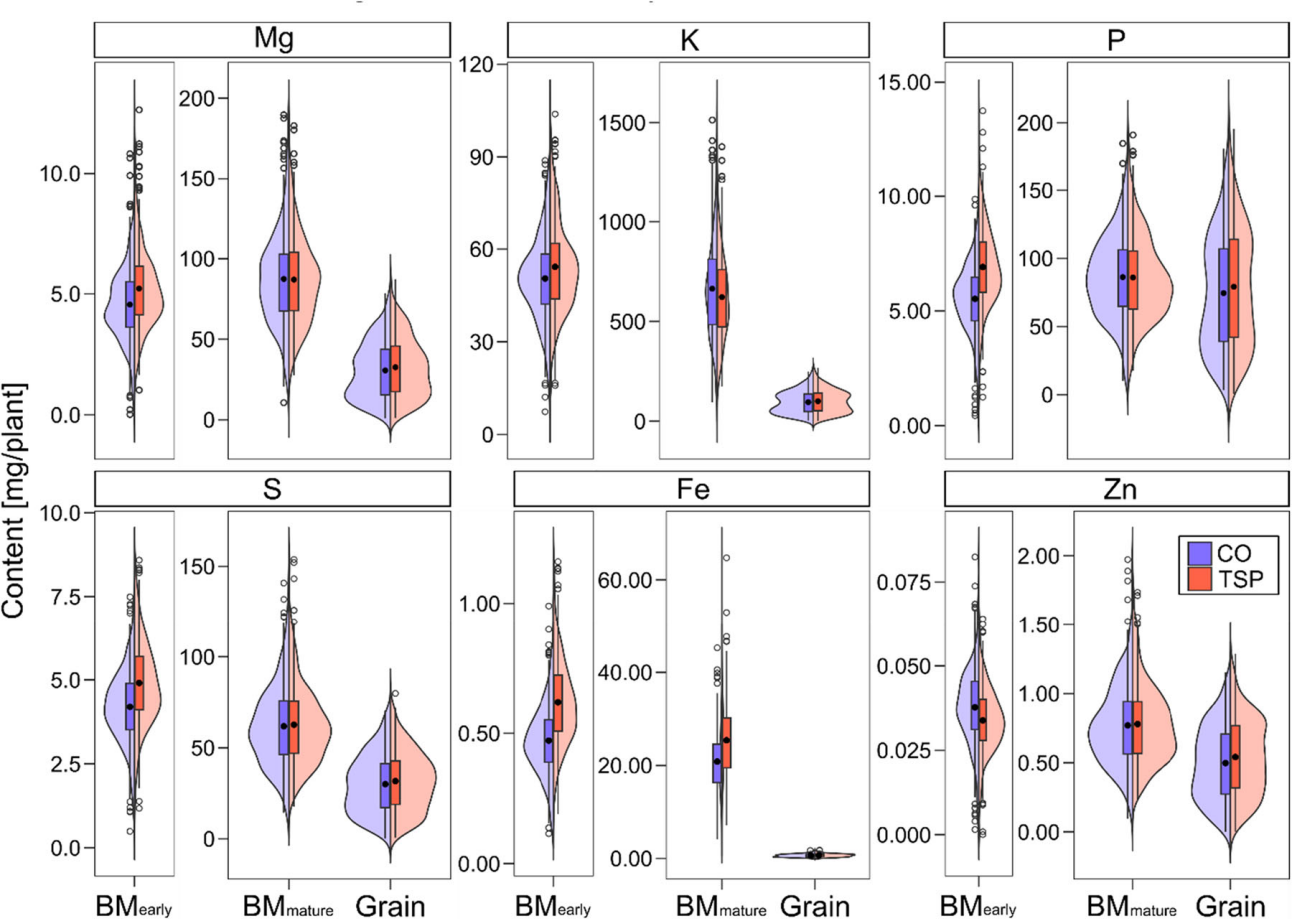
Distribution of mineral content across tissues and treatments. Boxplots and accompanying violin plots show the distribution of magnesium (Mg), potassium (K), phosphorus (P), sulphur (S), iron (Fe) and zinc (Zn) content. Data are presented separately for early biomass (BM_early_), late biomass (BM_mature_), and grain samples, and for the treatment with P starter fertiliser (TSP) and without (CO). [Color figure can be viewed at wileyonlinelibrary.com]

The most prominent effect on the mineral content, however, had the tissue. In general, the highest mineral content was in the late biomass tissue with sometimes only a fraction being in early biomass and grain samples (Figure 3; Supporting Information S1: Figure S5), an effect that can be attributed to the differences in dry weight yield. Only a few minerals such as Zn and P had comparable values between the biomass and grain tissue at maturity.

### 3.3 The Ionome Shows Strong Positive Correlations Across P Levels and Plant Tissues

**Figure 4:**
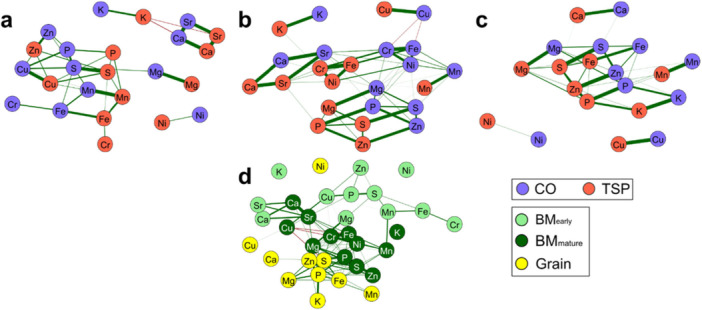
Network plots displaying mineral correlations under varying phosphorus (P) treatments and in different plant tissues. (a–c) Correlations between minerals in two P treatments, control and starter fertiliser for (a) early plant tissue (BM_early_), (b) mature plant tissue (BM_mature_) and (c) grain tissue. Correlations of the same mineral between treatments and different minerals within each treatment are represented by connecting lines. (d) Correlations between minerals across three plant tissues: early biomass, mature biomass and grain. The thickness and colour of the lines represent the strength and sign of the correlation: thick green lines indicate strong positive correlations, thin lines indicate weak correlations and thick red lines represent strong negative correlations. [Color figure can be viewed at wileyonlinelibrary.com]

A key objective of this study was to assess how the network of associations between minerals differs among the three plant tissues under contrasting P conditions. The high positive correlations within the ionome were more noticeable for the mineral content than for the concentration, but overall comparable between both. Out of the 153 correlations among minerals in BM_early_ and BM_mature_ and the 55 in grain, almost half or more were significantly positive (*p* < 0.05) within one tissue and treatment. Only rather low significant negative correlations were present between K and Sr (−0.32) as well as between K and Ca (−0.35) in BM_early_ and between Cu and Fe (−0.38) and Ni (−0.29) in BM_mature_. Nearly all macronutrients (P, K, Ca, S, and Mg) showed significant positive correlations between each other within the three tissue samples, except for K and Mg. In the correlation analysis across the two P starter fertiliser treatments, all minerals but Cr were significantly correlated between themselves in all tissues, indicating that the ionome within a specific tissue is consistent across different P soil levels. We also examined the correlations among elements across different tissues to identify patterns in element distribution and potential functional relationships. In the early and mature biomass samples, several elements (Ca, Mg, Mn, Sr, Cu) showed strong positive correlations, while correlations between biomass and grain were generally lower, though they were higher for biomass at maturity (S, P, Zn, Mn, Fe, Cu, Ni) (Figure 4).

### 3.4 Variation in Harvest Index and Mineral Composition Across Genetic Groups and Plant Tissues

**Figure 5:**
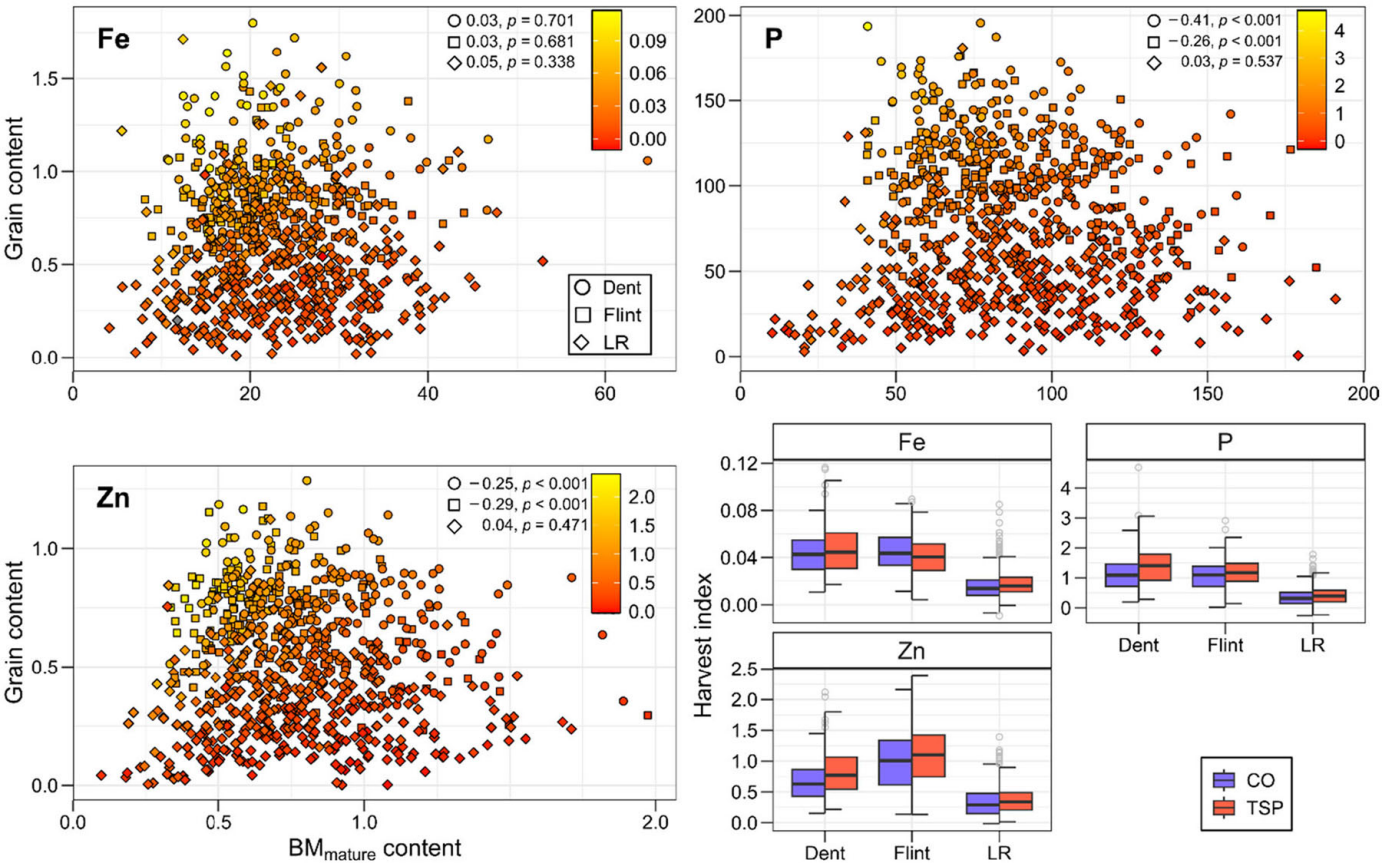
Relationship between biomass and grain mineral content, as well as the harvest index (HI) of minerals across genetic groups and treatments. (a) Correlations between late biomass (BM_mature_) and grain mineral content for iron (Fe), phosphorus (P), and zinc (Zn). Each data point represents a specific genetic group (denoted by shape), and its colour indicates the mineral harvest index, calculated as the ratio of grain to mature biomass mineral content. (b) Boxplots of mineral harvest indices across genetic groups (elite Dent, elite Flint and landraces) under two treatments, control (CO) and triple superphosphate (TSP). [Color figure can be viewed at wileyonlinelibrary.com]

The harvest index was calculated as the ratio of the mineral content in the grain and biomass at maturity and provides insights into the distribution of minerals within the plant. This harvest index showed a heritability of 0.62 for Ca under control treatment and up to 0.85 for Mn under P starter fertiliser application (Supporting Information S1: Figure S6). The genotypic effect had the largest singular impact, followed by genotype‐by‐environment interaction, while the genotype‐by‐treatment interaction variance was comparably small (Supporting Information S1: Figure S7). The starter fertiliser treatment influenced the harvest index values, which were generally higher under fertilised conditions. However, there was not always a significant difference. Not only had the treatment an effect, but also the minerals themselves varied in their translocation efficiency, as for example values where consistently higher for P than for Fe. Examining the subpopulations, elite Dent and Flint lines showed higher harvest index values than the six landraces, especially compared to the lines of the landrace ‘Gelber Badischer Landmais’ (Figure 5; Supporting Information S1: Figure S8). In addition to the harvest index, we also investigated the correlation between mineral content in grain and biomass at maturity. This was negative for elite Dent and Flint lines for minerals such as P and Zn, but almost zero for Fe and close to zero for the landrace lines for all three minerals (Figure 5).

**Figure 6:**
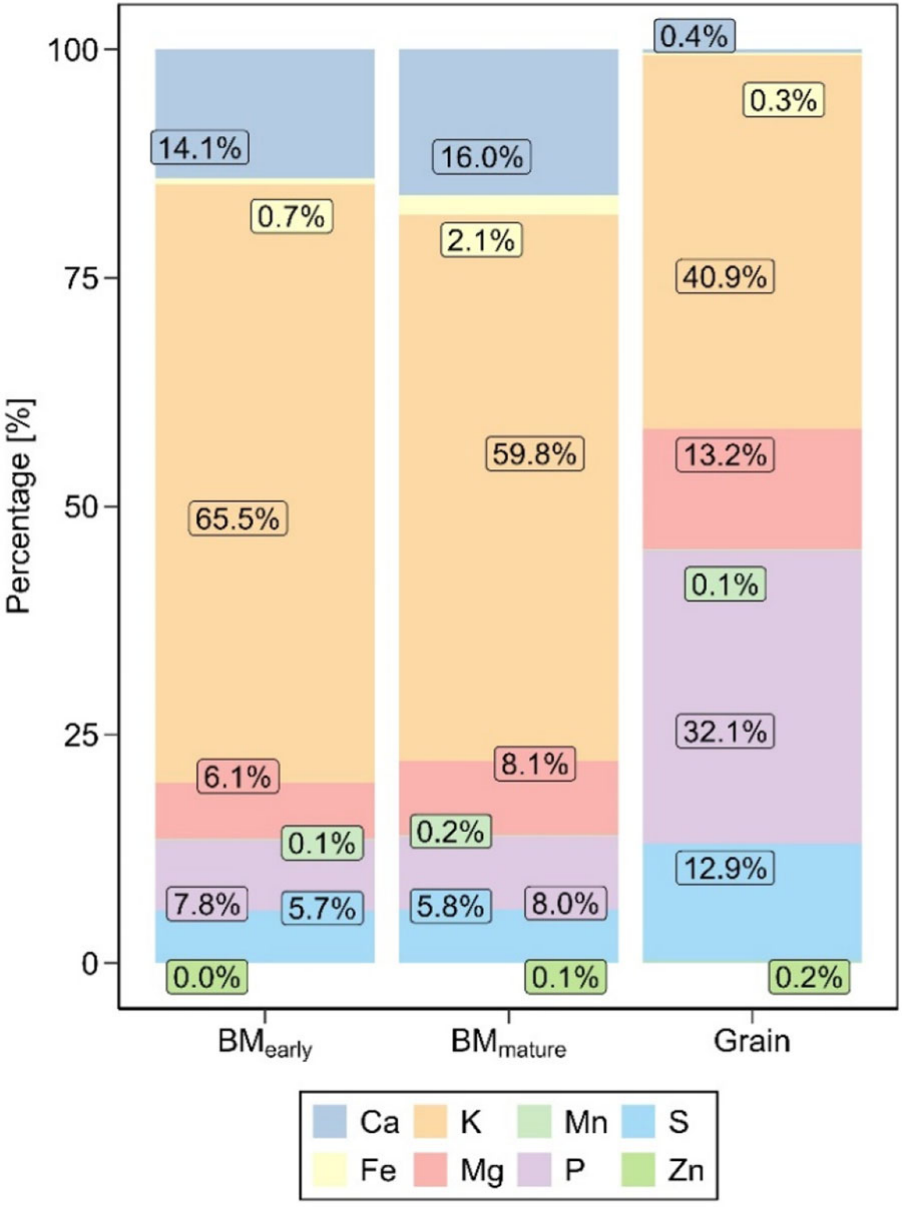
Relative composition of selected minerals within plant tissues. Displayed is the percentage composition of selected minerals in the control treatment (CO), shown for early biomass (BM_early_), late biomass (BM_mature_) and grain (Grain) samples. [Color figure can be viewed at wileyonlinelibrary.com]

Furthermore, we analysed the relative composition of eight selected minerals in each of the three tissues. The results revealed clear differences between the tissues, but only small differences between subpopulations and treatments (Supporting Information S1: Figures S9,S10). The most notable findings are the continuous decrease in the relative proportion of K from the young biomass to mature biomass and the grain and its consistently high content relative to other minerals across all three tissues. Additionally, a reduction of Ca concentration from around 14%–16% in early and late biomass tissue to only 0.4% in the grain, an increase of P by 24 percentage points from ~ 8% to ~ 32%, and an increase in Mg from ~ 8% to ~ 13% were observed (Figure 6).

### 3.5 Genome‐Wide Association Mapping Identified Significant Genetic Loci Associated With Mineral Traits

The genome‐wide association mapping was performed in the entire panel and for each subpopulation separately. Additionally, analyses were done individually for the two P treatments and for the three tissues. This approach enabled us to identify in which subset the QTL are active to reveal potential differences between them.

Putative QTL could be identified for all mineral traits, ranging from two for Cr to 55 unique QTL for Mn concentration. Most QTL were identified for the complete panel (303) followed by the landraces (108), while for the elite Dent and Flint lines 74 and 80 QTL were detected, respectively. The average proportion of explained genotypic variance of all the QTL was 13.18%, with 53 QTL explaining more than 30%.

**Figure 7:**
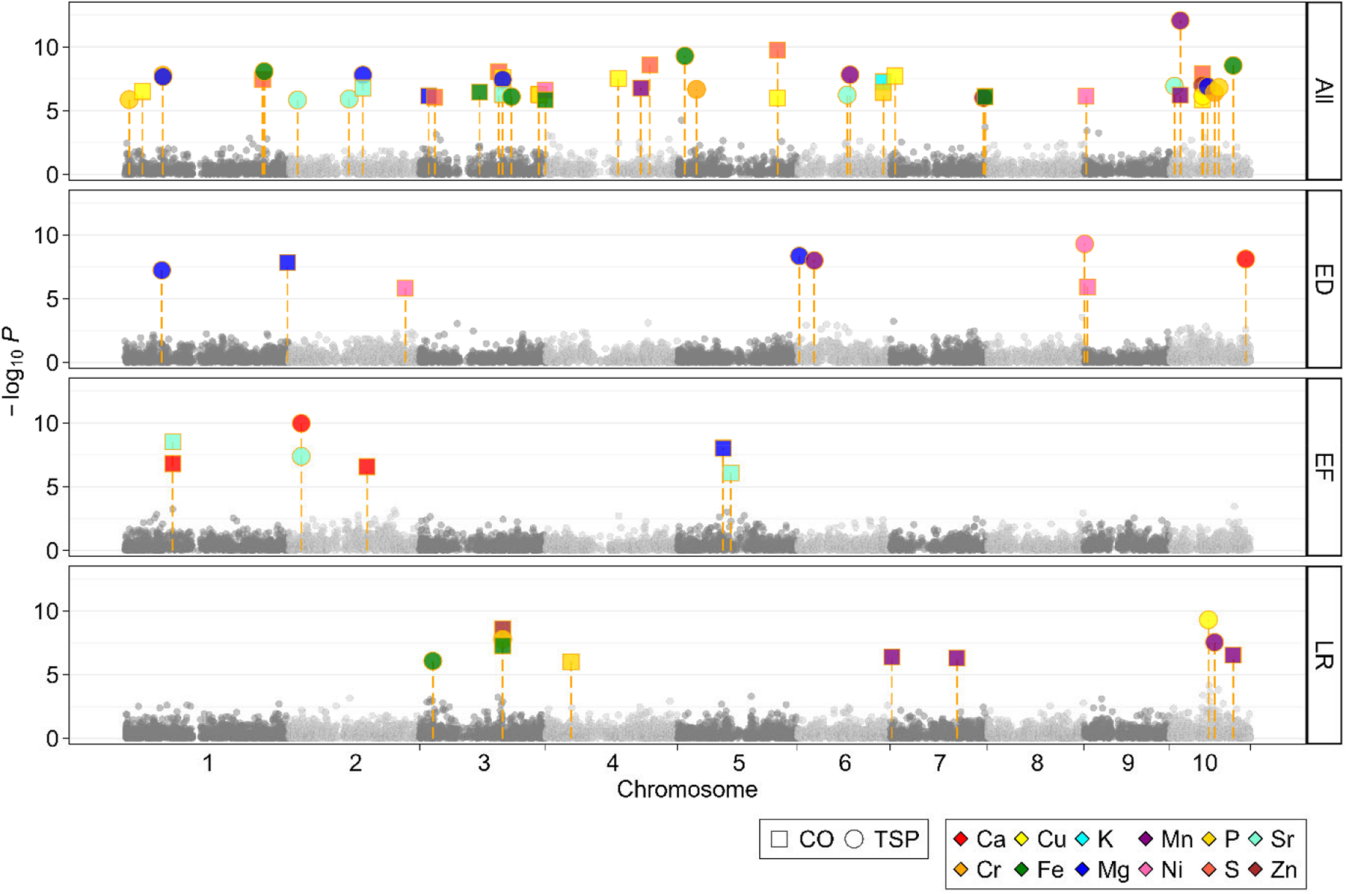
Manhattan plot showing SNP associations of mineral content traits in early biomass. Results are shown for the whole panel (All), the elite Dent (ED) lines, elite Flint (EF) lines, and the landrace (LR) lines. Highlighted dots indicate significant SNPs distinguished by their trait‐specific colour and treatment shape. All significant SNPs are plotted, while for nonsignificant SNPs every 100th position is shown. [Color figure can be viewed at wileyonlinelibrary.com]

QTL were defined as overlapping if they were located within 50 kbp of each other. This revealed common QTL across different minerals, subpopulations and treatments. Specifically, the proportion of common QTL between the two starter fertiliser treatments was found to be 11.41% (63 out of 552 QTL). This overlap was particularly pronounced in the whole diversity panel, especially for Ca content and Cu, Mg, Mn, and S concentration. A rather small number of common QTL was identified between the three subpopulations, with 5.43% being common between them. Common QTL across tissues were rare, often reflecting connections between different treatments and subpopulations. Overall, 79 QTL were found to have pleiotropic effects on two or more traits, most of them in the whole panel for BM_mature_ tissue (Figure 7, Supporting Information S1: Figures S11–S15).

## 4 Discussion

### 4.1 Plant Breeding Has the Potential to Improve Mineral Content

In this study, we addressed the variation in mineral uptake and allocation and their complex relationship. Assessing mineral elements in a diverse maize panel and across tissue types as well as developmental stages offers a more comprehensive understanding of how the ionome is controlled at a genetic, tissue, and environmental level.

For nearly all the studied minerals, the genotypic variation significantly contributed to the observed phenotypic differences, which is a common observation for ionomics data (Baxter et al. 2012; Gu et al. 2015; Yang et al. 2018). In line with this, the heritability of the mineral traits was generally medium to high (Supporting Information S1: Figure S1). As exceptions, Fe and Cr showed lower heritabilities in certain tissue‐treatment combinations, which could be caused by contaminations of the samples by soil (Yang et al. 2018). While heritability estimates showed differences depending on tissue type and treatment, our results of a generally high degree of genetic control of these traits are consistent with previous studies in rice (Garcia‐Oliveira et al. 2009; Yang et al. 2018) and Arabidopsis (Baxter et al. 2012). These results suggest that the ionome is mostly under genetic control, which opens the possibility for selecting more favourable genotypes and improving these mineral traits.

The different environments also had a substantial effect on the phenotypic variation (Figure 1), which is in accordance with previous studies in maize (Gu et al. 2015), wheat (Zhang et al. 2010) and potato (Abebe et al. 2012). These results show that the mineral content is directly influenced by the soil in which the crop is grown and thereby by the inherent variability in soil characteristics across different locations (Khasawneh et al. 1980). Also, climatic conditions can influence mineral uptake, as for example rainfall affects nutrient solubility. For several minerals, we observed a higher environment variance for the early developmental stage than at maturity. The soil composition and nutrient availability are critical during the early stages of plant growth, as young plants are often more sensitive to environmental conditions and nutrient availability. This was, for instance, shown for P in barley (Green et al. 1973) and maize (Barry and Miller 1989), and for Zn in rice (Slaton et al. 2005), where early deficiency had a bigger impact on growth and yield. Plants at this stage are establishing their root system, and differences in soil composition and climate can affect root development (Richner et al. 1996; Ferguson et al. 2016; Hobson et al. 2023), making plants more susceptible to the variations in their immediate environment. We also observed a significant genotype‐by‐environment interaction variance for some trait‐tissue combinations, which is in agreement with previous results (Brown et al. 2013; Gu et al. 2015). A stable expression of minerals across environments would be advantageous for breeding and for optimal variety choice by the farmers at their location. In practice, if the relevance of the genotype‐by‐location interaction is known, breeders can select for varieties with either broad or specific adaptation. The principal component analysis based on the ionome data showed no distinct groups as most of the minerals, including noxious or non‐essential elements such as Sr, displayed significant positive correlations, irrespective of the genetic background (Supporting Information S1: Figure S4). Similar results were observed for Arabidopsis (Baxter et al. (2012), maize (Baxter et al. 2013) and Chinese cabbage (*Brassica rapa* L. ssp. *pekinensis*) (Wu et al. 2008). Consequently, when breeding specifically for mineral accumulation and content, it is possible to simultaneously improve multiple minerals, but it is also crucial to consider these correlations to avoid the accumulation of undesired minerals. Finding no significant negative correlations was somewhat unexpected, as different minerals can compete for plant root uptake sites on cell membranes by the nonselective uptake of several metal cations into the root, like Cu and Mg (Jiang et al. 2007) or Cu and Zn in wheat landraces (Akcura and Kokten 2017). The correlations among minerals between the tissues showed that the phenotypes are relatively repeatable between tissues, more so between early and mature biomass tissue than between biomass tissue and grains.

Notably, if biofortification is a breeding goal, plant breeders will have to take care to not only increase yield across locations but also to focus on relevant nutrients such as Fe and Zn. In wheat, for example, Shariatipour et al. (2021b) reported a substantial co‐localisation of QTL for grain yield and grain Fe content and consequently, by combining multiple QTL regions through marker‐assisted selection, their analysis pointed to the potential to concurrently enhance micronutrient levels, grain quality, and yield. Biofortification through plant breeding can enhance the nutritional value of common foods, offering an affordable, sustainable and long‐term solution to increase micronutrient intake (Bouis et al. 2014). It must also be noted, that we evaluated the line per se performance and further research is required to investigate the inheritance of the ionome in a hybrid background. Nevertheless, the observed genotypic variance and heritability results show that breeding can boost micronutrient levels, leading to a valuable improvement of the nutritional status of crops.

### 4.2 The Genetic Material Influences the Ionome

In general, the observation by Bender et al. (2013) and Gregorio (2002), that nutrient uptake has increased along the breeding history of maize and other crops, was also seen in our study, as the mineral content was higher in kernels of modern breeding lines than in landraces (Figure 2). It is reasonable to assume that a long selection process and changed crop management strategies increased grain yield but also shaped the mineral uptake traits. Our study also showed that elite lines exhibited a more stable performance across environments (Supporting Information S1: Figure S3).

In a study on P‐use efficiency in Flint maize breeding lines, Li et al. (2021) argue that P‐use efficiency and P‐acquisition efficiency declined over the last decades due to reduced root systems and the ability to exploit external P. Their study focused, however, on seedlings until 3 weeks after germination. It is possible that the uptake of nutrients is postponed in modern genotypes to provide an elongated vegetative phase with a prolonged photosynthetic activity even in late development (Yang et al. 2018). Flint landrace lines were also higher in nutritional content in our study in early development, perhaps due to their fast youth development compared to Dent lines (Figure 2). This extensive variation among the subpopulations, especially elite Dent and Flint lines, could be beneficial in future hybrid crosses to improve nutrient content.

### 4.3 Variation in Phosphorus Fertilisation Can Affect the Ionome

A review of the effects of nutrient antagonism and synergism on yield and fertiliser‐use efficiency showed that most interactions between minerals are synergistic, among others, due to the beneficial impact of macronutrients on root reductase activity and phytosiderophore production (Rietra et al. 2017). Consistent with this, we observed a significantly higher P, Mn, Ca, Cu and K uptake when applying P starter fertiliser under field conditions. Phosphorus availability in the soil is known to affect the uptake of other minerals by improving root growth and thereby increasing the uptake efficiency of various minerals. This was shown in the study of Fageria et al. (2014), where the nutrient uptake of the minerals mentioned above was significantly increased with P addition in legume cover crops, and conversely lower P conditions for rice led to decreased concentrations of some elements (Yang et al. 2018). However, other studies found contrasting results, that P fertilisation actually reduced mineral concentration, and assigned this to a ‘dilution’ effect of minerals by increased growth under P fertilisation (Davis 2009; Uygur and Şen 2018).

Interestingly, despite the beneficial increase in various mineral concentrations with P fertilisation, this practice also leads to a notable decrease in Zn content. On a molecular basis, P is thought to influence Zn uptake by inhibiting the movement from root to shoot and decreasing its availability in the soil (Haldar and Mandal 1981; Zhu 2001), although the exact mechanisms are unknown (Brian J. Alloway 2008). The additional application of P starter fertiliser in our study led to the Zn content being 10.27% lower in young maize tissue compared to the control. This observation agrees with studies on corn silage on sandy soil (Drissi et al. 2015) and rice grown in a greenhouse setting (Haldar and Mandal 1981). This may also lead to P uptake efficient genotypes being repressed in their Zn uptake efficiency, especially in more deficient soil. Consequently, disproportionate P fertilisation on Zn‐deficient soils could hinder the efforts of Zn biofortification. Dietary micronutrient deficiency like Zn is prevalent in many low‐ and middle‐income countries (Gupta et al. 2020). Not only does P hinder the accumulation of Zn, it is also mainly stored as phytate in the grain. This storage form has a strong affinity to several dietary trace elements and can inhibit the bioavailability of Zn (Schlemmer et al. 2009).

In contrast, for minerals whose homoeostasis remained unaffected by the starter fertiliser, the availability of P in the soil does not seem to alter the underlying regulatory mechanisms. Agricultural sustainability will depend on reducing fertiliser inputs, and plant breeding will need to achieve this goal without compromising yield and quality. Our study revealed that reduced P fertilisation compared to the standard practice could alter the ionome composition, which in turn affects kernel quality in terms of essential and harmful elements.

### 4.4 Substantial Variation in Mineral Composition and Translocation

One of our objectives was to evaluate the mineral composition in vegetative and generative tissue to assess the translocation between these plant organs. Looking at the temporal aspect of nutrient accumulation, our results showed that plant mineral content tends to increase as the plants mature (Figure 3; Supporting Information S1: Figure S5) (Yang et al. 2018). The mineral content in late biomass is primarily influenced by two factors: nutrient uptake after silking and the remobilisation of nutrients accumulated before, the latter specifically affecting the grain content (Karlen et al. 1988; Kichey et al. 2007; Bender et al. 2013). The high mineral need in late developmental stages could be partly due to greater dry matter production during late reproductive development, especially for high‐performing genotypes. This phenomenon could be even more pronounced in genotypes with the functional stay‐green trait, which extends the photosynthesis period (Bender et al. 2013). According to Chen et al. (2016), the mechanisms by which minerals are taken up and distributed among plant organs during development are organ‐specific and depend on both the environment and the mineral itself. This can explain the difference in mineral content between the elite Dent and the landraces in the early biomass tissue as well as between elite Flint and the landraces in the grain (Figure 2). The observed negative correlations between grain and mature biomass for P and Zn in Dent and Flint lines suggest a trade‐off in nutrient allocation between vegetative biomass and grains. This may indicate that these two elite heterotic groups prioritise reproductive output, likely as a result of selection for high grain yield. The lack of this correlation in landrace lines could reflect the lower selection pressure in these populations. The absence of any significant correlation for Fe across all subpopulations implies that high Fe content in biomass does not guarantee high Fe translocation to grains and that grain content likely depends more on direct uptake during grain filling than on remobilisation from vegetative tissues (Figure 5). This could be due to the low mobility of Fe between plant organs compared with P (Hocking 1994; Miller et al. 1994), resulting in its retention in vegetative tissues rather than active translocation to grains, irrespective of biomass levels. The harvest index for P was higher than for Fe and Zn (Figure 5), which leads to a similar conclusion, that a larger proportion of the total P is accumulated in the grain than in the biomass compared to the other minerals. This indicates that P is highly mobile in plants. Its essential task in energy transfer and nucleic acid synthesis, which are required in developing grains, may prioritise P translocation to grains to support reproductive development.

The nutrient partitioning analysis confirmed this significant increase in the grain's relative percentage of P compared to the maize plant's vegetative part (Figure 6). Studies in maize and herbs have shown that plants continue to relocate P from above‐ground plant tissue into grain during the late growing season (Hanway 1962a, 1962b; Fenner 1986), and probably also take up further P from the soil, as in other studies the loss from the above‐ground biomass did not align with the amount relocated into the grain (Sayre 1948). In contrast, compared to other minerals, the relative percentage of K decreased consistently from young biomass to grain. This indicates a shift in the proportion of K relative to the total mineral content as the plant matures. Previous studies on maize have shown that K uptake is completed by the silking stage (Karlen et al. 1987) and subsequently, the plant either depletes its K or the accumulation slows down (Sayre 1948; Hanway 1962b).

We further investigated the nutrient translocation with the mineral harvest index that contrasts the mineral content in grain and in the biomass at maturity, and thereby offers insights into the distribution and thus the translocation of minerals within the plant. For crop plants, it is crucial to consider not just the mineral content in the harvested parts, but also in the non‐harvested biomass that remains on the field. Notably, we observed a moderate to high heritability (Supporting Information S1: Figure S6) and a significant genotypic variance for the mineral harvest index (Supporting Information S1: Figure S7), showing that it is possible for breeding to alter the distribution of minerals within the plant. Our results revealed that for several minerals the mineral harvest index changes with the addition of P starter fertiliser (Figure 5, Supporting Information S1: Figure S8). The vegetative parts of the plant retained less minerals under P starter fertilisation than in the control without this additional fertiliser. Consequently, we and other researchers found an increase in harvest index values of the minerals (Dordas 2009). In wheat field studies, Batten (1986) reasoned that lower harvest index values in the treatment without additional fertiliser could be due to accumulation of P in vegetative tissue. There are divergent opinions on which mineral harvest index is advantageous, which are, for example, presented in the review of Batten (1992) about P efficiency in wheat. On the one hand, concerns have been expressed about nutrient removal from the soil for crops with a high mineral harvest index (Meelu et al. 1979; Tan et al. 2005; Masters et al. 2016). In regions with decreasing fertility levels in the soil, higher yielding genotypes with greater harvest index values will have a high nutrient removal and will need higher fertiliser applications to maintain nutrient levels in the soil. Low harvest index values for minerals would retain more minerals on the field, unless the straw is used as animal feed, where it would be more beneficial for livestock but also removed from the field. On the other hand, low mineral harvest index values for P have been shown to not increase yield and could even lead to reduced seed size (Batten and Wardlaw 1987; Batten and Slack 1990) and less robust plants if the seed is used for cultivation (Bolland and Baker 1988; Conceição et al. 2019). Thus, future breeding can target the mineral harvest index to tailor varieties to the specific needs of the agricultural system, the environment and the expectations regarding the product, but may sometimes have to balance the different needs and thus the distribution within the plant.

### 4.5 Genome‐Wide Association Mapping Gives Insights Into the Complex Genetic Architecture of the Ionome

The inheritance of the mineral concentration and content was found to be, for the most part, of a quantitative nature (Figure 7; Supporting Information S1: Figures S11–S15), which corroborates previous findings (Vreugdenhil et al. 2004; Wu et al. 2008; Blair et al. 2009; Garcia‐Oliveira et al. 2009). Although a high positive correlation was observed between most minerals (Figure 4), which might suggest a shared genetic basis as observed in rice (Garcia‐Oliveira et al. 2009) and Arabidopsis (Shariatipour et al. 2021a), only a few QTL were common between minerals, for example between Ca and Sr content. These two minerals exhibit similar atomic radii and closely related chemical properties (Coelho et al. 2017). Indeed, their similarities extend beyond soil and plant uptake behaviour (Drouet and Herbauts 2008; Watanabe et al. 2016), with Sr also serving as a tracer for Ca (Storey and Leigh 2004). Wu et al. (2008) also found correlations between Mg, Ca and Sr, but few overlapping QTL. An analysis of cationic mineral content in seeds of Arabidopsis found common QTL between K and Ca as well as between K/Ca/Mn, but also mineral‐specific QTL (Vreugdenhil et al. 2004). The same was true for shoot Ca and Mg concentrations in *Brassica oleracea* (Broadley et al. 2008). This may in part be due to the large number of QTL with small effect involved in mineral uptake and allocation, so that a common QTL is identified for one mineral but not for the other. Nevertheless, these results suggest that in addition to common QTL, the minerals are controlled by trait‐specific QTL, allowing to modulate one mineral without affecting others. Co‐locating QTL between minerals can be due to a pleiotropic effect of the QTL or due to close genetic linkage of distinct QTL each affecting only one trait. In case of the same allele being advantageous, common QTL can enable a faster and easier simultaneous selection of the target minerals (Shariatipour et al. 2021a; Shariatipour et al. 2021b). However, as the colocalized QTL between Ca and Sr illustrate, such common QTL can also include harmful minerals, that would be co‐selected alongside the desired mineral.

The low overlap of QTL, as well as the higher correlation between mineral concentrations within the tissues than between them, suggest an in part tissue‐specific regulation of mineral concentration and potentially a substantial environmental impact shaping the observed variation. Consequently, it also means that the ionome at an early developmental stage is not predictive for the mineral content in the grain. Hence, researchers and breeders should adhere to the tissue of interest when characterising the ionome.

To identify potential candidate genes, we extracted genes within 50 kbp of the significant SNPs by considering their annotation and the functions of homologous genes. A promising candidate was found close to a marker (AX‐90533180) significant in the whole panel for the P concentration in the grain under P fertilised conditions. This gene, encoding *Inositol‐pentakisphosphate 2‐kinase*, was also identified in Arabidopsis (Gulabani et al. 2022) and wheat in the phytic acid biosynthesis pathway, and in wheat, silencing of this gene resulted in a larger quantity of free phosphate in the grain and an increase in Fe and Zn content (Aggarwal et al. 2018; Whitt et al. 2020). As mentioned, phytic acid is an important variable in optimising biofortification as it can hinder the bioavailability of other micronutrients. Another interesting candidate gene, a *glutathione peroxidase*, was found near a marker (AX‐90533377) identified in the whole panel as well as the in the landrace lines for Cu in mature biomass tissue in both treatments. In rice seedlings, glutathione minimised Cu toxicity by decreasing its uptake (Mostofa et al. 2014), and Cu treated Arabidopsis seedlings showed similar behaviour (Smith et al. 2004). Close to the same marker, a *heavy metal ATPase*, or *Putative ATP dependent copper transporter* was present, which in Arabidopsis functions in Cu detoxification of roots (Andrés‐Colás et al. 2006; Whitt et al. 2020), and in transporting the mineral in the roots and other organs (Deng et al. 2013). Furthermore, a yellow stripe‐like transporter was found close to the marker AX‐90566196 associated with Cu content in mature biomass tissue in the elite Dent lines. This gene is thought to be involved in Fe and Cu translocation, remobilizing the minerals from mature leaves to flowers and seeds (DiDonato et al. 2004; Jean et al. 2005; Whitt et al. 2020). A further candidate gene related to Mn transport was found in the whole panel for mature biomass tissue (AX‐91340746). This metal transporter *Natural resistance‐associated macrophage protein‐like* (*Nramp*) enables Mn entry into root cells (Sasaki et al. 2012; Castaings et al. 2021; Bozzi and Gaudet 2021).

## 5 Conclusions

In this study, we investigated the ionome in a large diversity panel of maize. Our results revealed substantial genotypic variation and an effect of the environment, as well as an interaction between the two. Also the mineral composition and the translocation showed substantial variation. Moreover, we found that the ionome changes in response to the phosphorus fertilisation regime. The results of the genome‐wide association mapping highlight the quantitative nature of the mineral concentration and content, but also revealed targets for further research and selection in breeding. While the number of identified QTL is limited, the identification of key loci for traits like zinc and iron content offers valuable insights for improving the mineral profile of maize through marker‐assisted selection, though further research into QTL stability across diverse environmental conditions is needed. Collectively, our results illustrate the variation and the dynamics of the ionome as a basis toward its targeted design.

## Conflicts of Interest

The authors declare no conflicts of interest.

## Supporting information

Supporting Figures.

## Data Availability

The data that support the findings of this study are available from the corresponding author upon reasonable request.
