# Supplementary material for: Genetic Variation and Quantitative Trait Loci Analysis of the Maize Ionome in Response to Phosphorus Fertilisation
Source: Plant Cell Environ. 2025 Sep 3;48(12):8727–42. doi: 10.1111/pce.70174 (PMC12586916; doi:10.1111/pce.70174)
Supplement: Supplementary file 1 — Supporting Figures. [file PCE-48-8727-s001.docx]

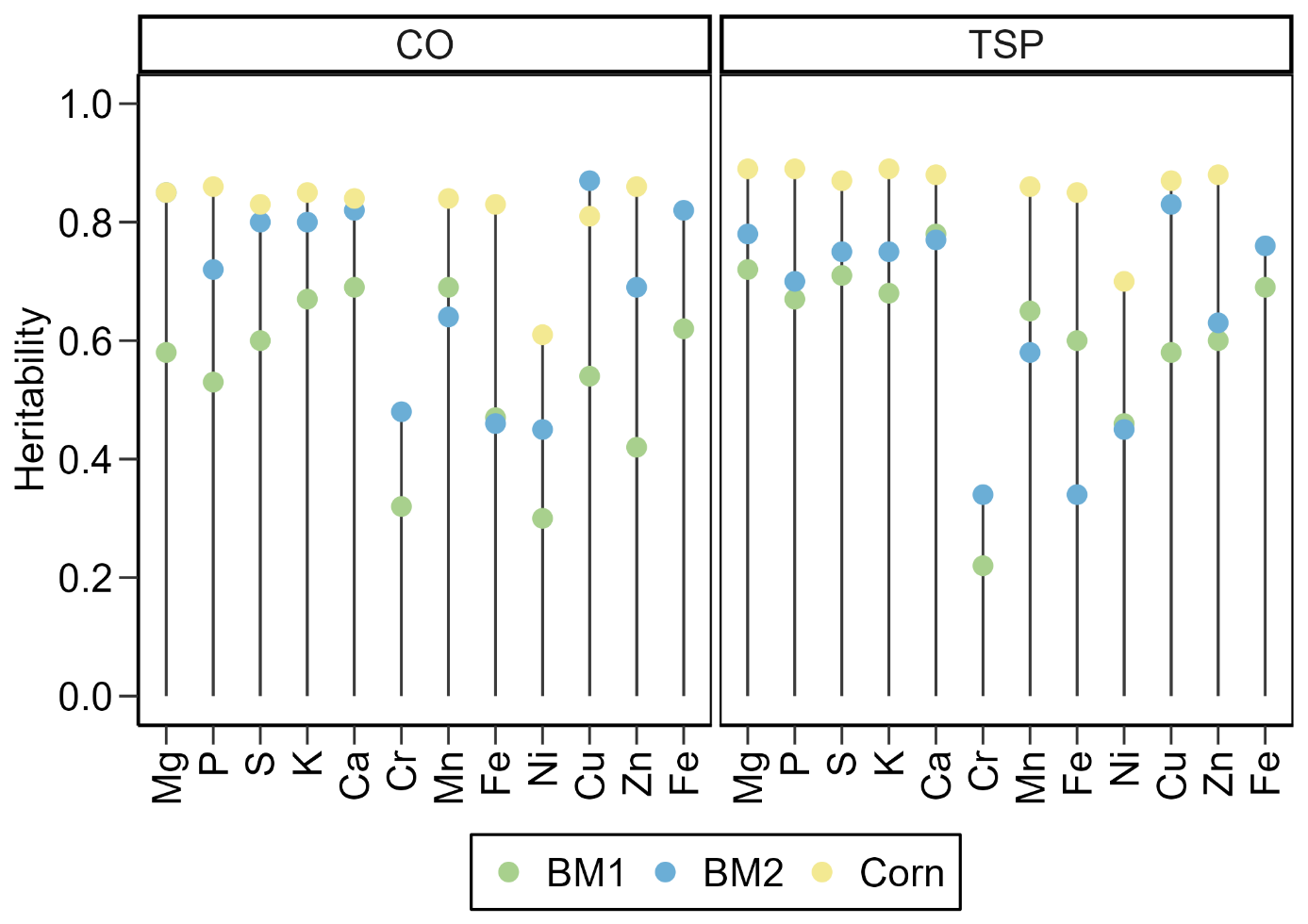


**Supp Figure 1 *Heritability of Mineral Content Across Tissues and Treatments.*** *Heritability estimates for the mineral contents, with each point representing a different tissue (early biomass = BM_early_, late biomass = BM_mature_, Grain). The plot is faceted by two treatments with (TSP) and without phosphorus starter fertilizer (CO).*


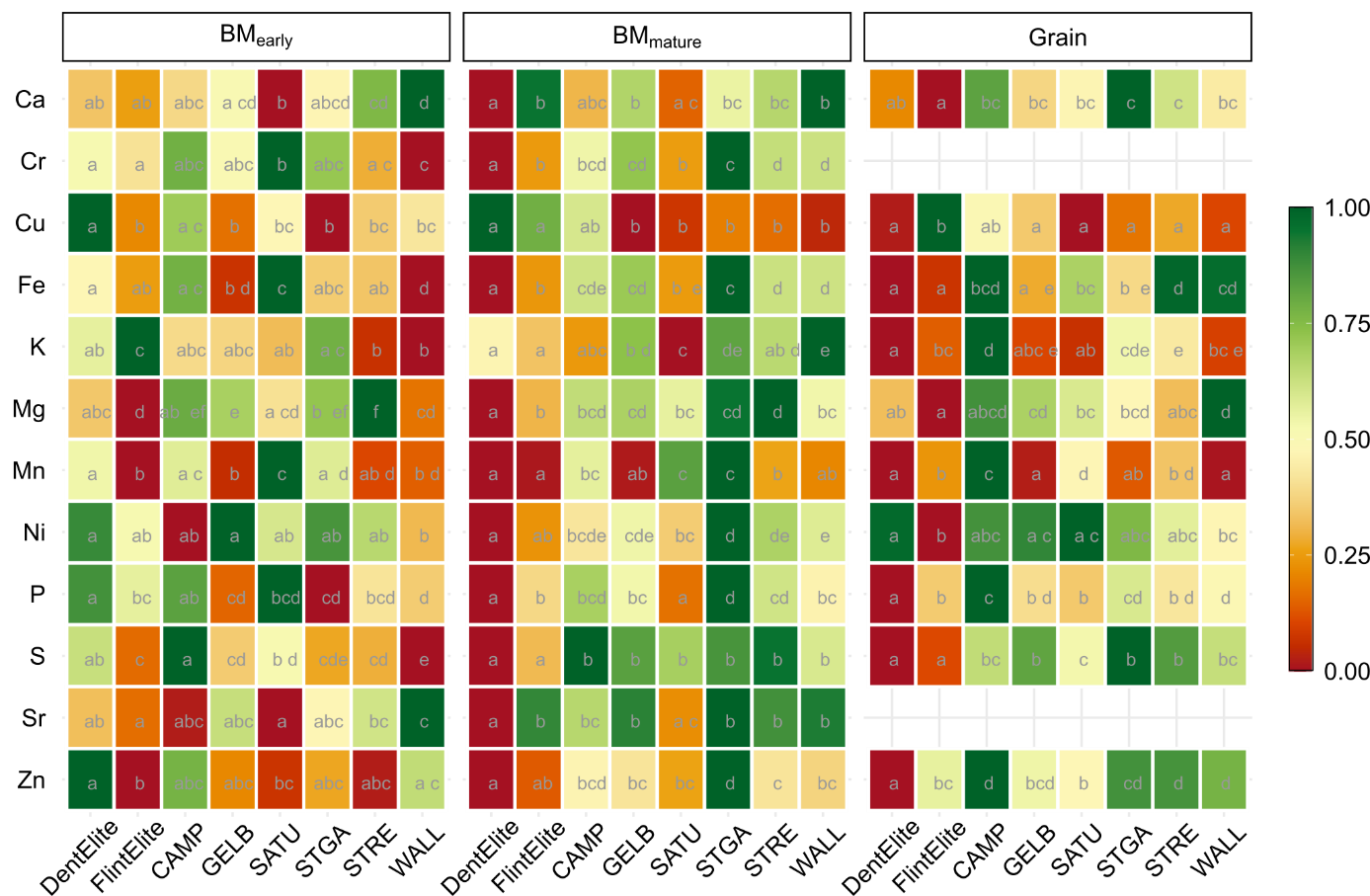


**Supp Figure 2 *Ionome variation between genetic groups and tissues.*** *Displayed is the standardized mean mineral concentration for the Dent and Flint elite lines and for each of the landraces in the control treatment, separated by tissue (BM_early_, BM_mature_, Grain). The color indicates the mean standardized value. Letters within the panels denote statistically significant differences by analysis of variance based on a linear model fit among genetic groups for a given mineral.*


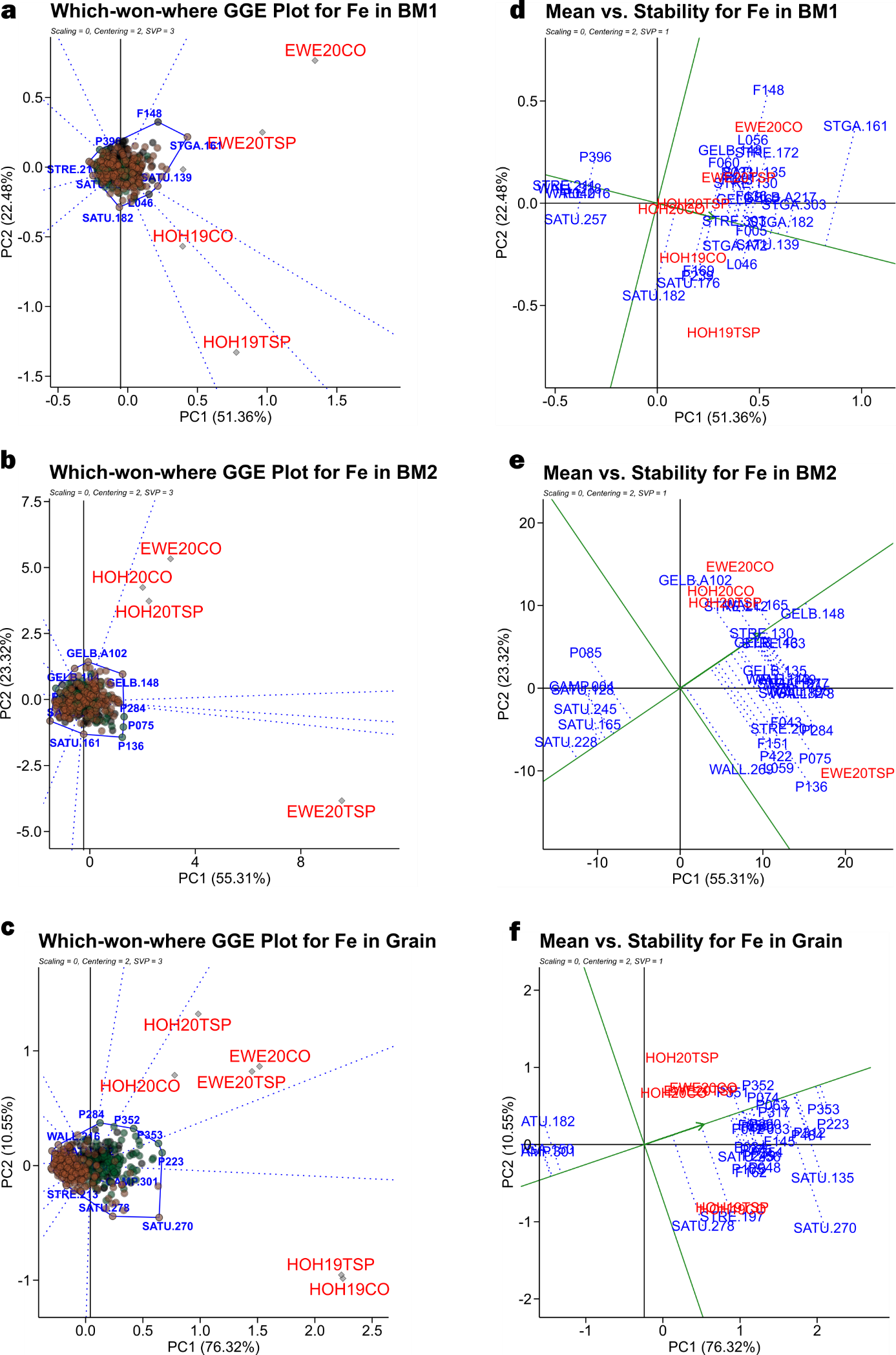


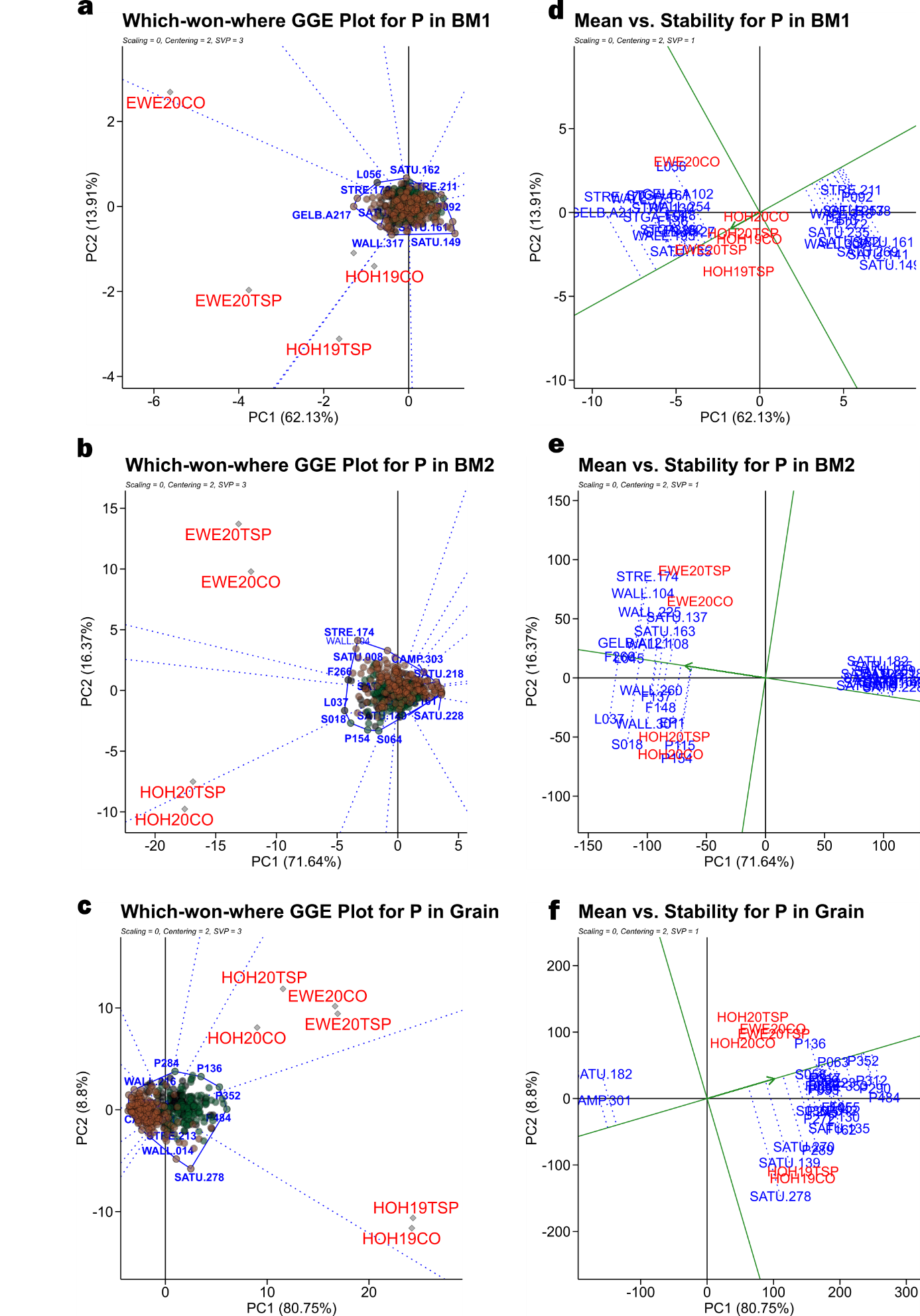


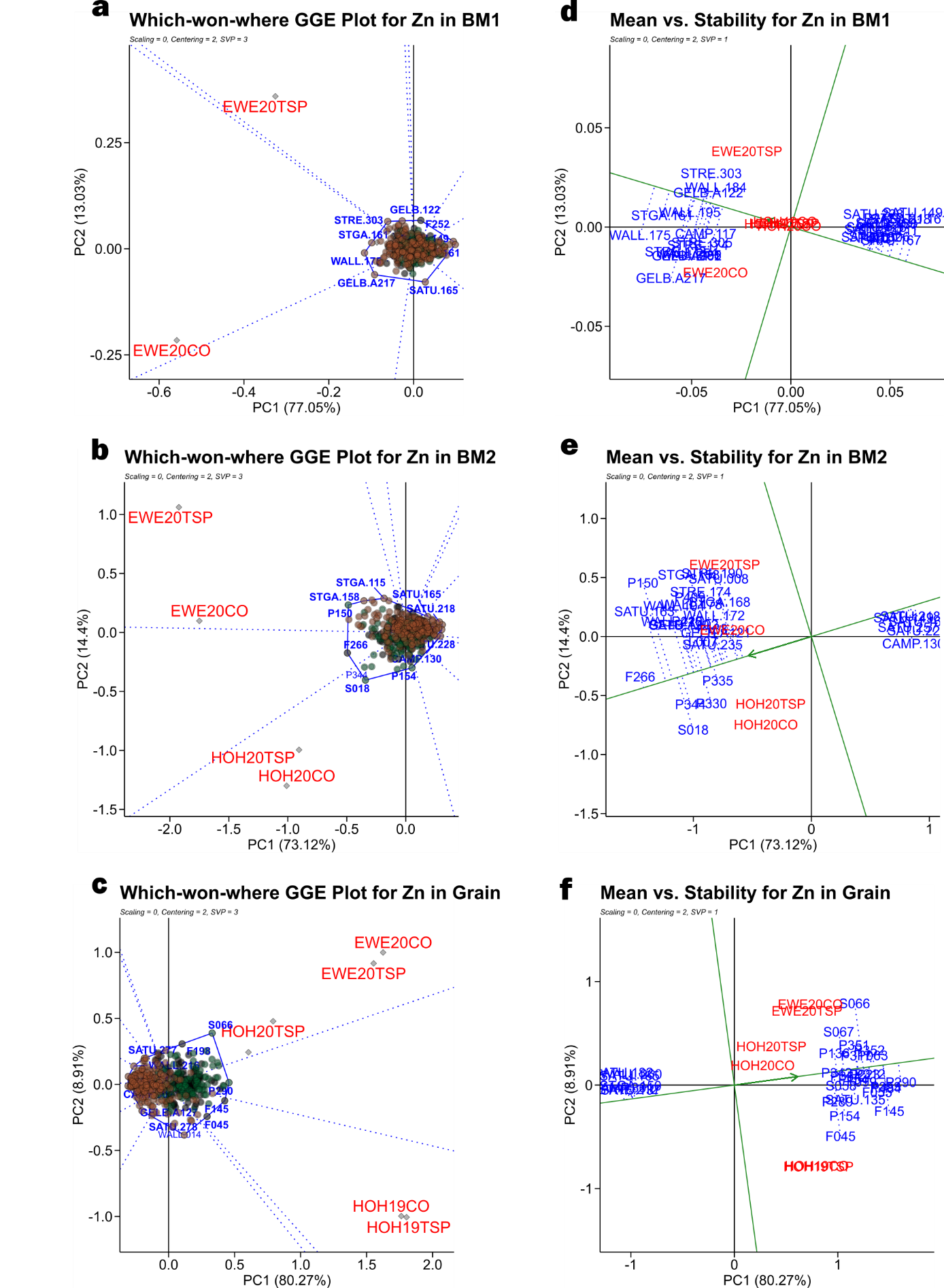


**Supp Figure 3 *Location-centered GGE biplots*** *for iron (Fe), phosphor (P) and zinc content (Zn) in early biomass (BM1), biomass at maturity (BM2) and grain tissue.* ***Which-won-where biplots (a-c)*** *with polygons connecting the most responsive genotypes; sectors indicate environment groupings where vertex genotypes perform best. Environments are shown in red. Genotypes are colored by group: landrace lines (brown), elite Dent lines (green), and elite Flint lines (black).* ***Mean vs. Stability biplot (d-f)*** *dashed blue lines indicate variation in performance across environments (longer = less stable). Genotypes are projected onto a green axis representing mean performance, with the arrow pointing toward higher values. Environments are shown in red.*


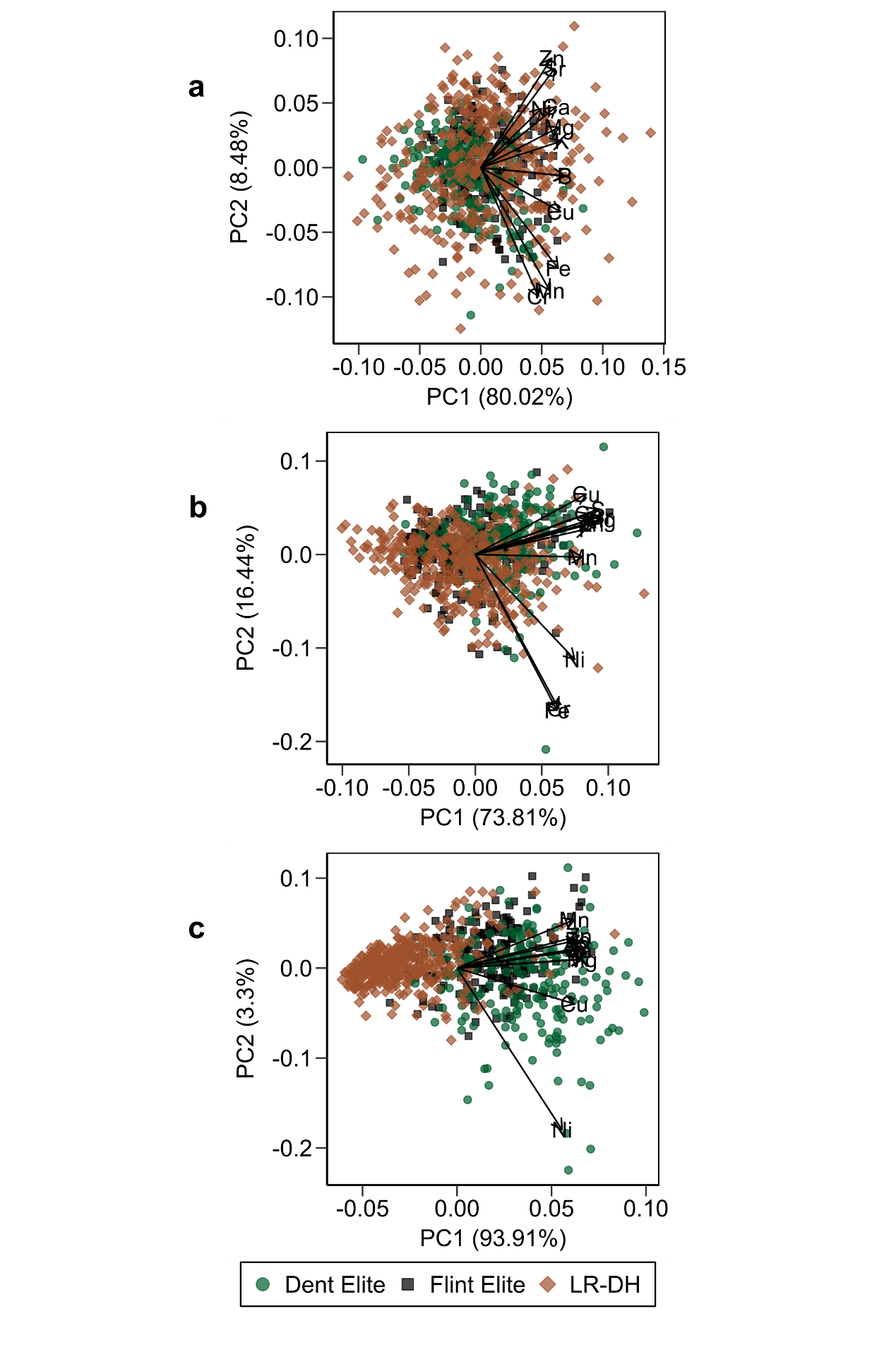


***Supp Figure 4 Principal component analysis (PCA) of mineral traits in different tissues.*** *Results are shown without Phosphorus starter fertilizer. The genotypes are assigned to their material group as Dent Elite, Flint Elite and Landraces (LR).* ***a*** *PCA for early biomass,* ***b*** *in mature biomass and* ***c*** *for grain samples.*


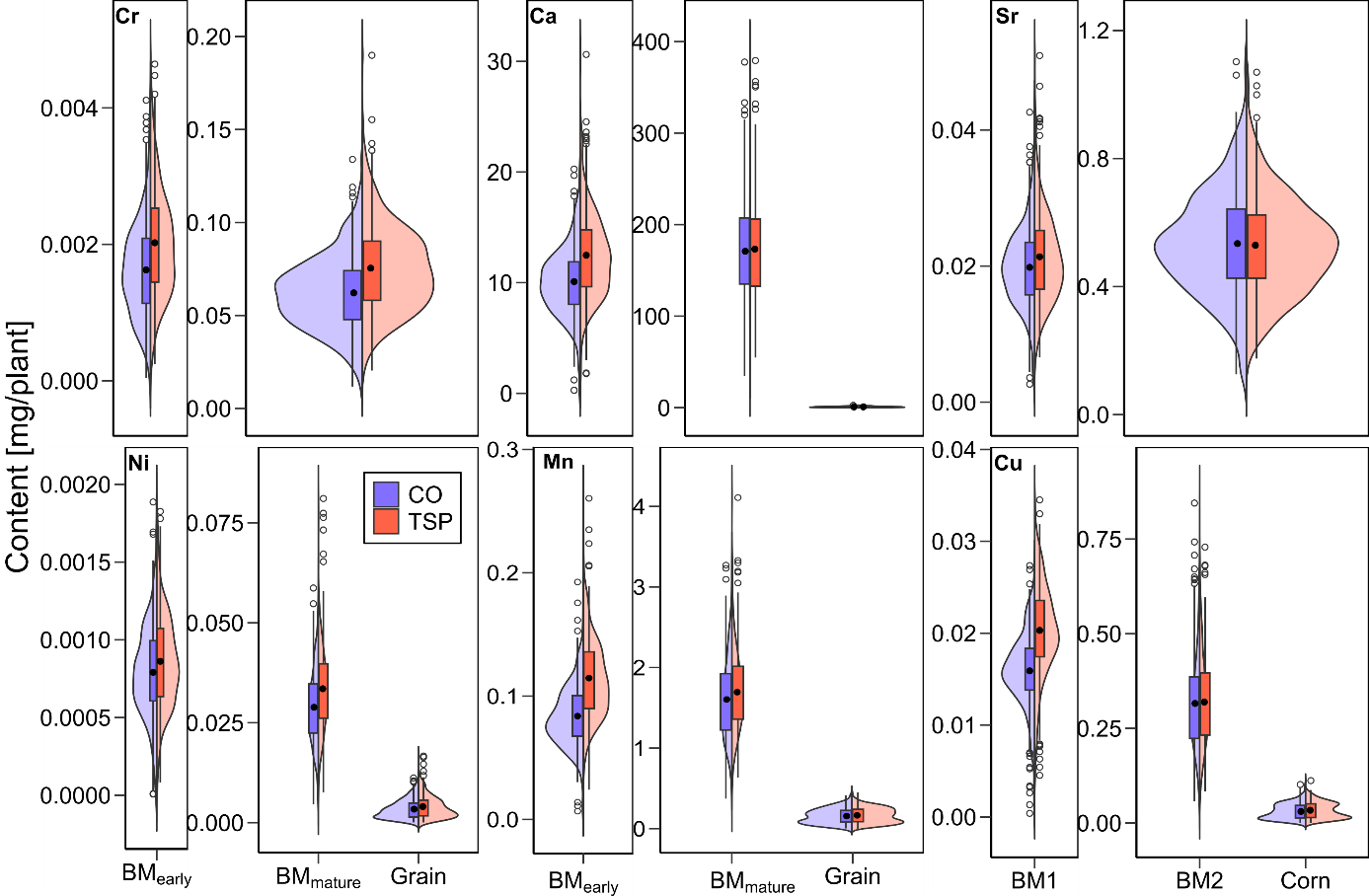


**Supp Figure 5 *Distribution of Mineral Content [mg/plant] Across Tissues and Treatments.*** *Boxplots and accompanying violin plots show the distribution of Chromium (Cr), Calcium (Ca), Strontium (Sr), Manganese (Mn), Copper (Cu) and Nickel (Ni). Data are presented separately for early biomass (BM_early_), late biomass (BM_mature_), and grain samples, measured with P starter fertilizer (TSP) and without (CO).*


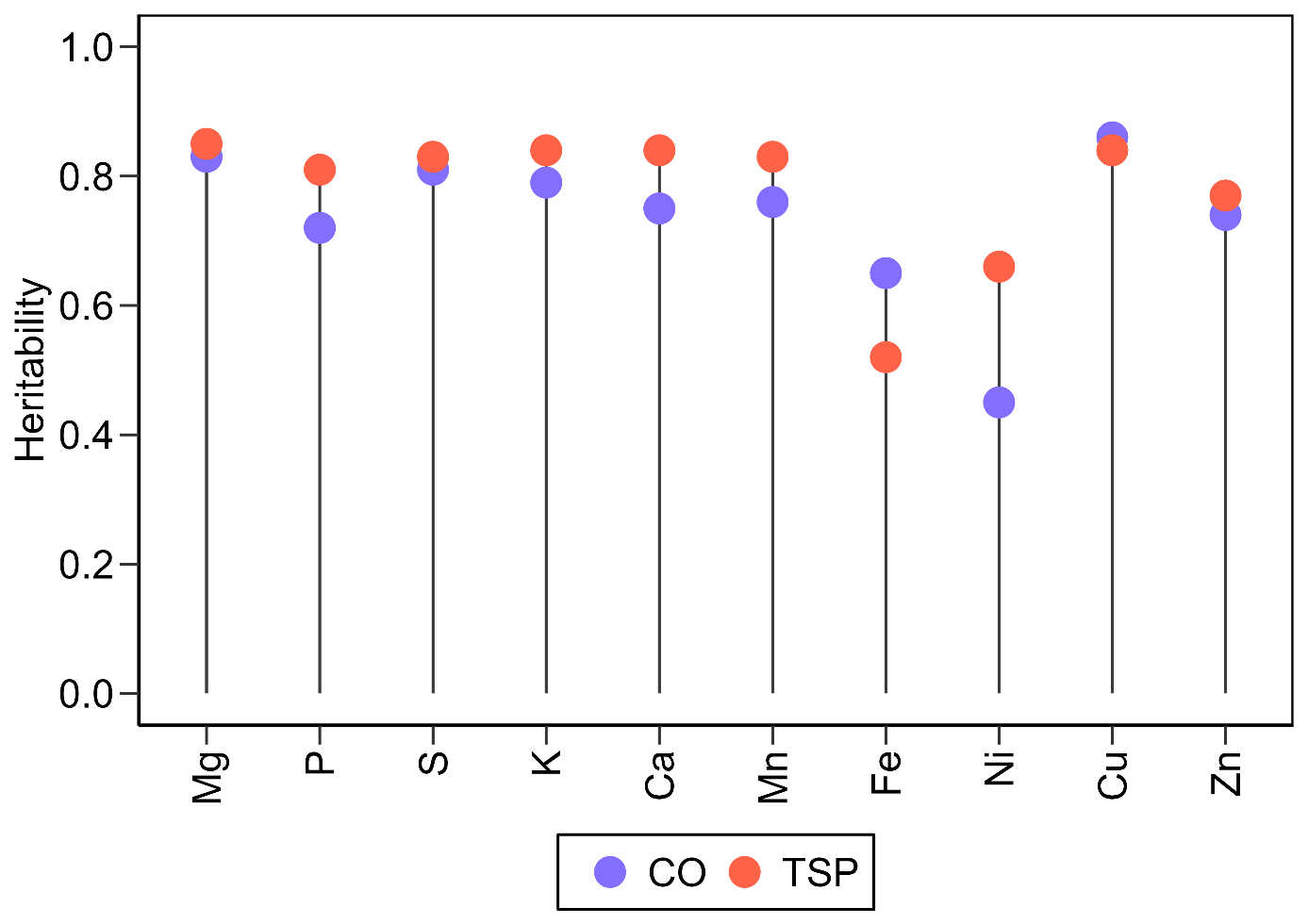


**Supp Figure 6 *Heritability of Mineral Harvest Index Across Tissues and Treatments.*** *Heritability estimates for the mineral harvest index, with each point representing a different treatment (CO = without Phosphorus starter fertilizer; TSP = with Phosphorus starter fertilizer). Minerals shown are Magnesium (Mg), Phosphorus (P), Sulphur (S), Potassium (K), Calcium (Ca), Manganese (Mn), Iron (Fe), Nickel (Ni), Copper (Cu), Zinc (Zn).*


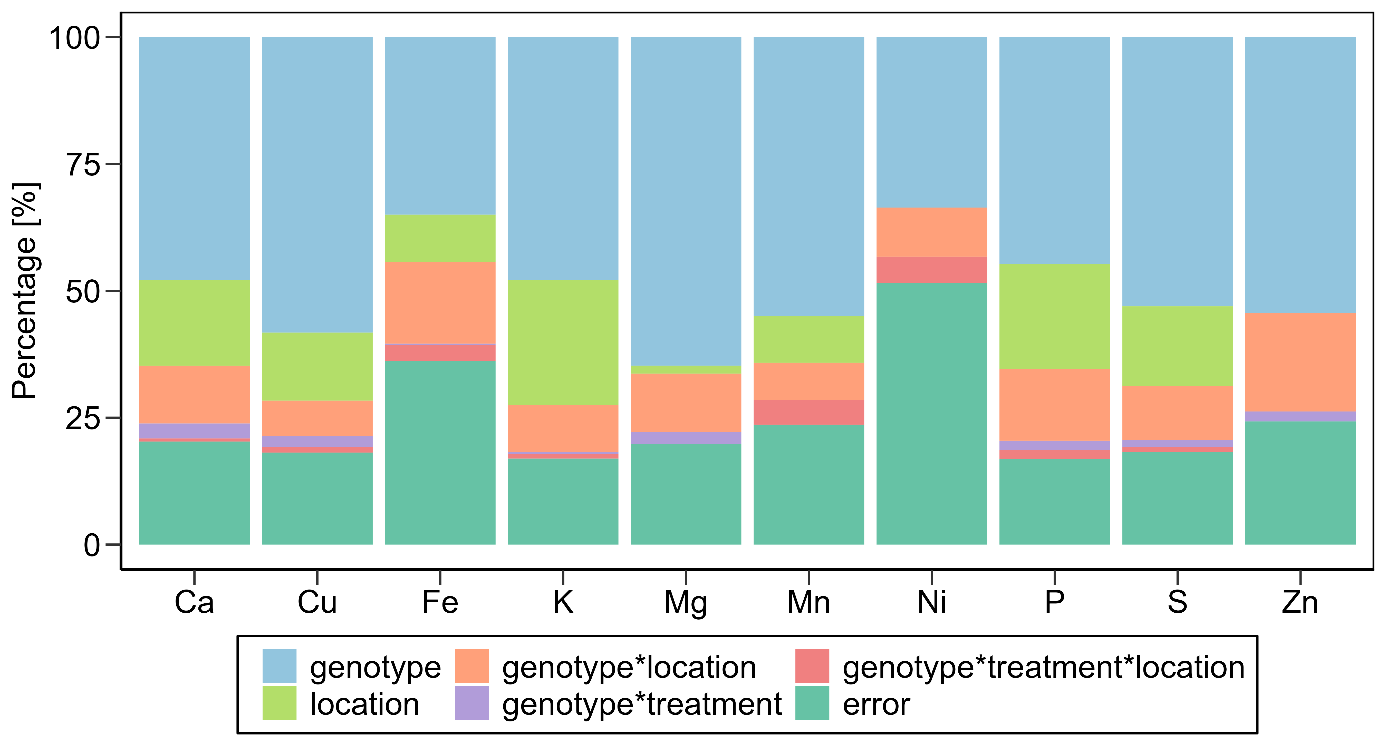


**Supp Figure 7 *Percentage Contribution of Variance Components for Mineral Harvest Index****. Analysis of the percentage contributions of variance components for mineral harvest index across treatments. Minerals shown are Calcium (Ca), Copper (Cu), Iron (Fe), Potassium (K), Magnesium (Mg), Manganese (Mn), Nickel (Ni), Phosphorus (P), Sulphur (S), and Zinc (Zn).*


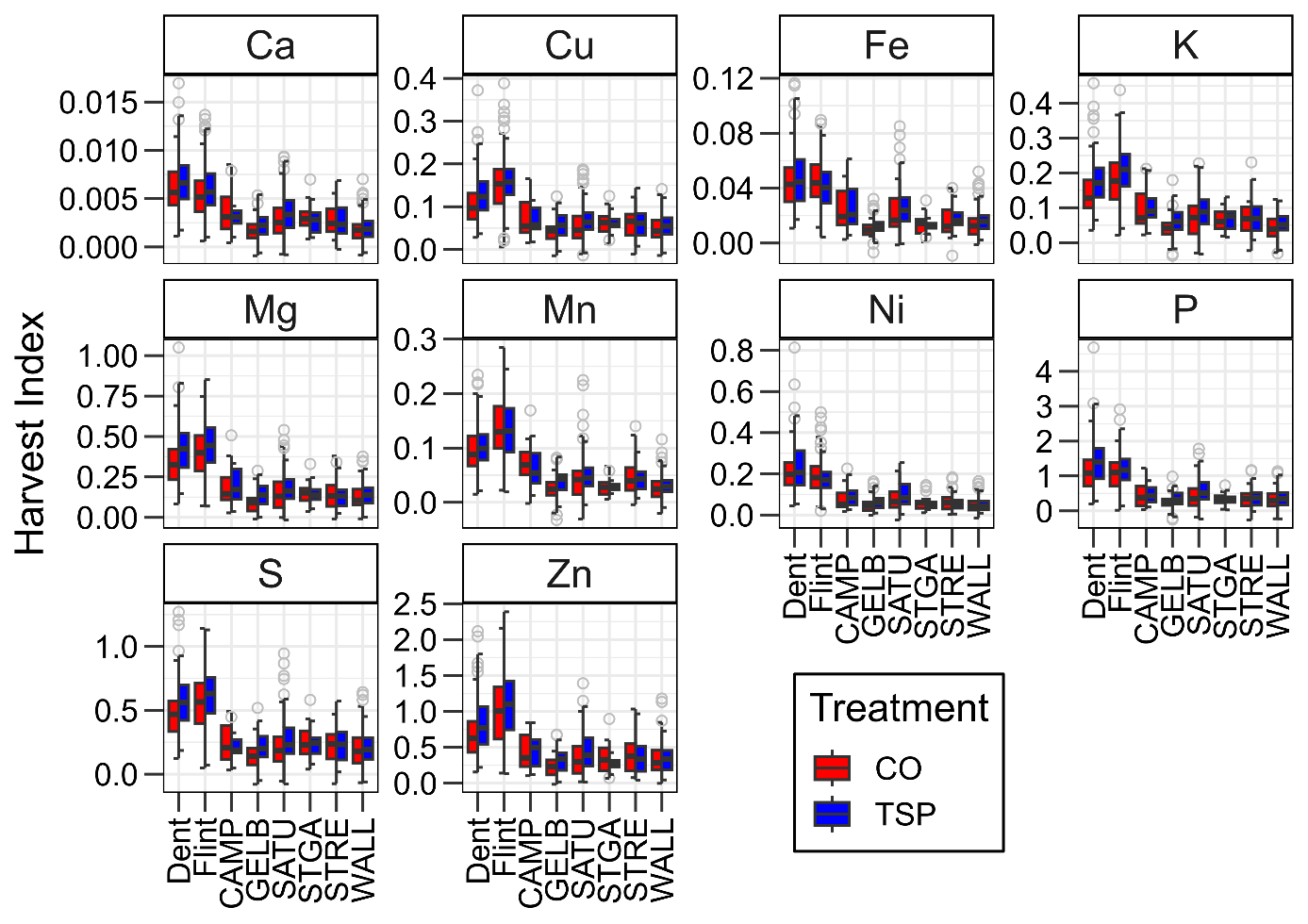


**Supp Figure 8 *Variation of Mineral Harvest Index between Minerals and treatments.*** *Minerals shown are Calcium (Ca), Copper (Cu), Iron (Fe), Potassium (K), Magnesium (Mg), Manganese (Mn), Nickel (Ni), Phosphorus (P), Sulfur (S) and Zinc (Zn). Boxplots show trait distributions without (CO) or with (TSP) starter fertilizer for each subpopulation.*


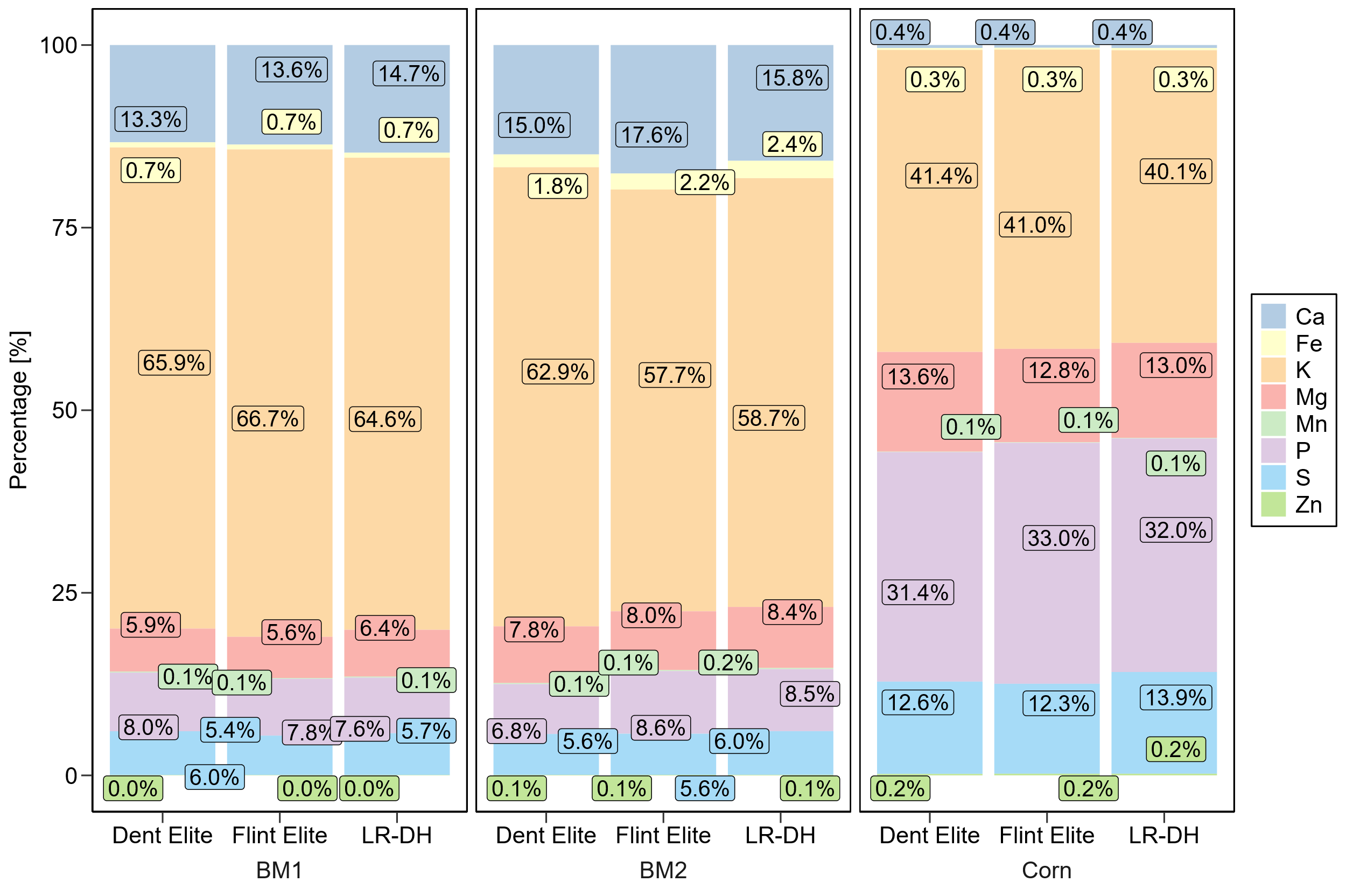


**Supp Figure 9 *Composition of selected minerals within plant tissues.*** *Displayed is the percentage composition of selected minerals in each subpopulation (LR-DH = doubled haploid landraces lines), shown for early biomass (BM_early_), late biomass (BM_mature_) and grain (Grain) samples.*


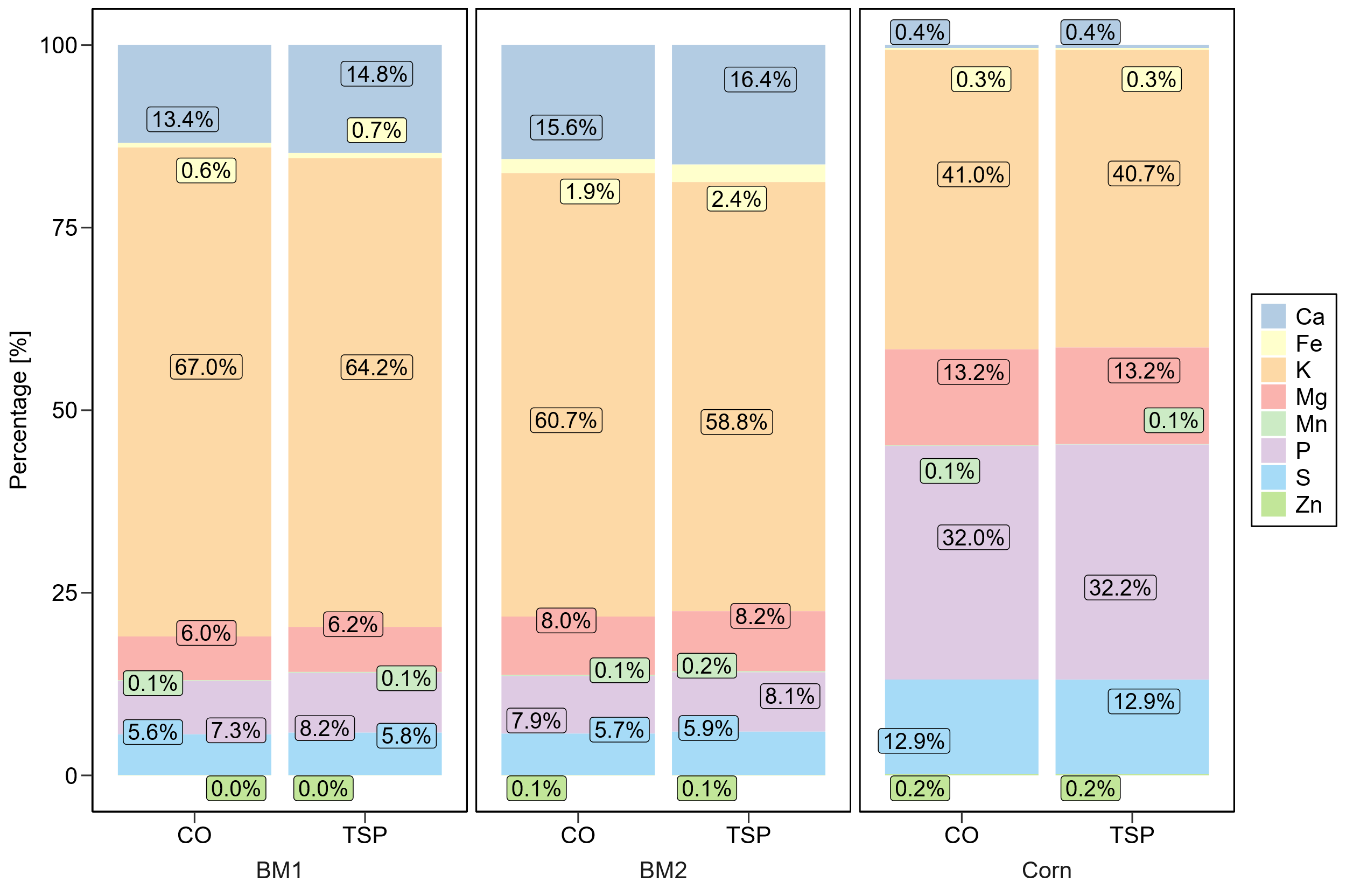


**Supp Figure 10 *Composition of selected minerals within plant tissues.*** *Displayed is the percentage composition of selected minerals in each treatment (CO = control; TSP = fertilizer treatment), shown for early biomass (BM_early_), late biomass (BM_mature_) and grain (Grain) samples.*


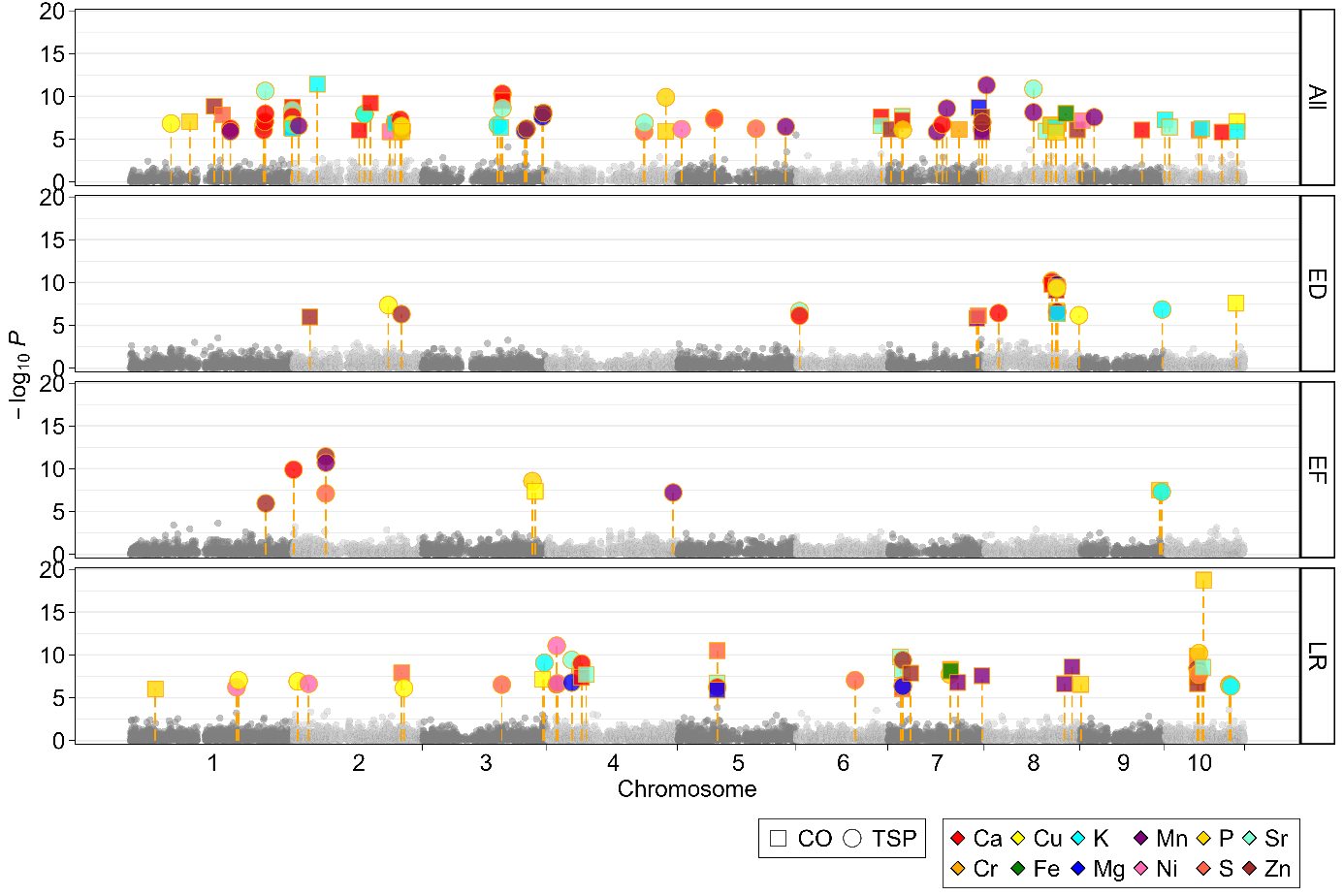


**Supp Figure 11 *Manhattan plot showing -log10(p-value) of SNP associations of mineral content traits in late biomass.*** *Highlighted dots indicate significant SNPs distinguished by their trait-specific color and treatment shape. All significant SNPs are plotted, while for non-significant SNPs every 100th position is shown.*


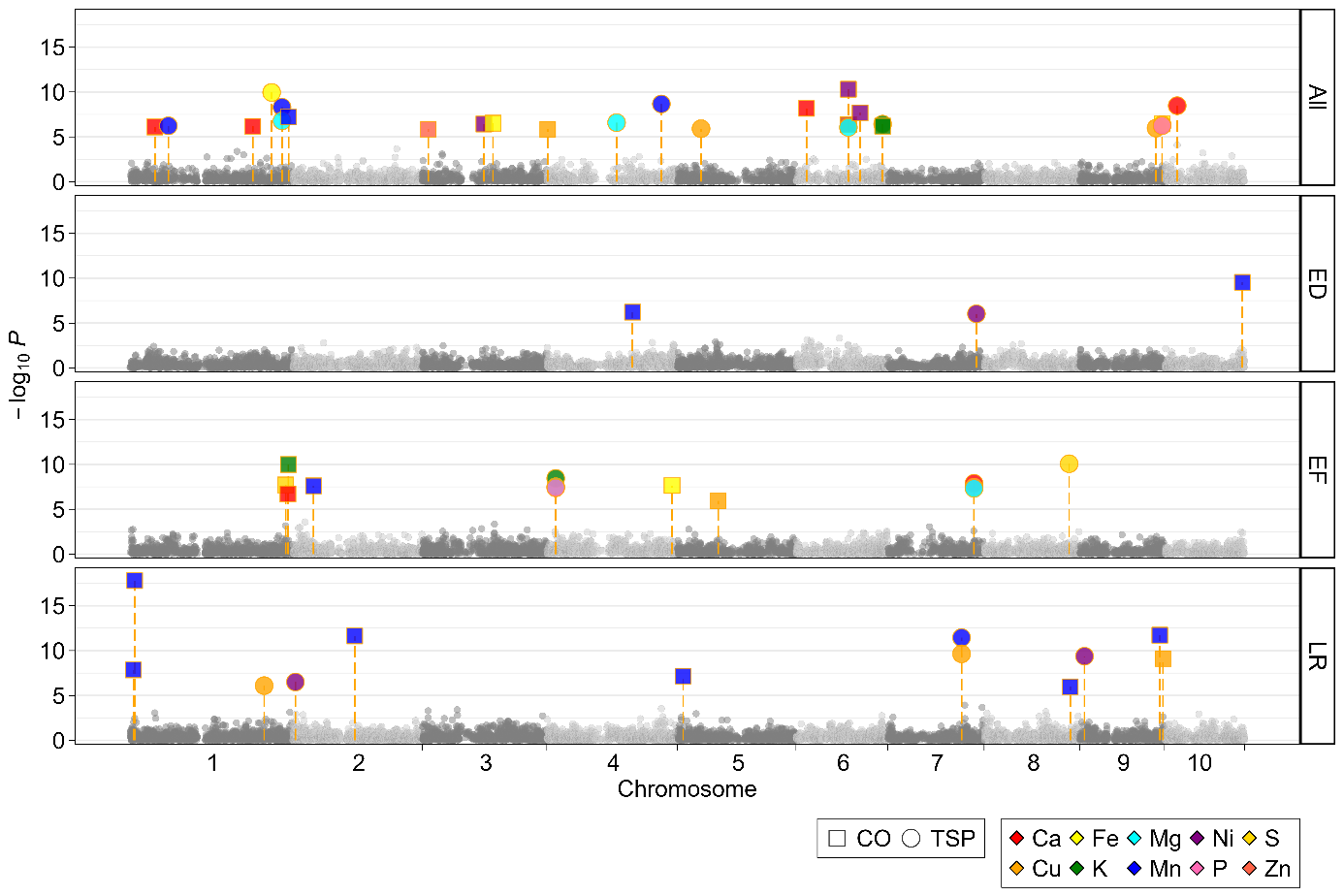


**Supp Figure 12 *Manhattan plot showing -log10(p-value) of SNP associations of mineral content traits in the grain.*** *Highlighted dots indicate significant SNPs distinguished by their trait-specific color and treatment shape. All significant SNPs are plotted, while for non-significant SNPs every 100th position is shown.*


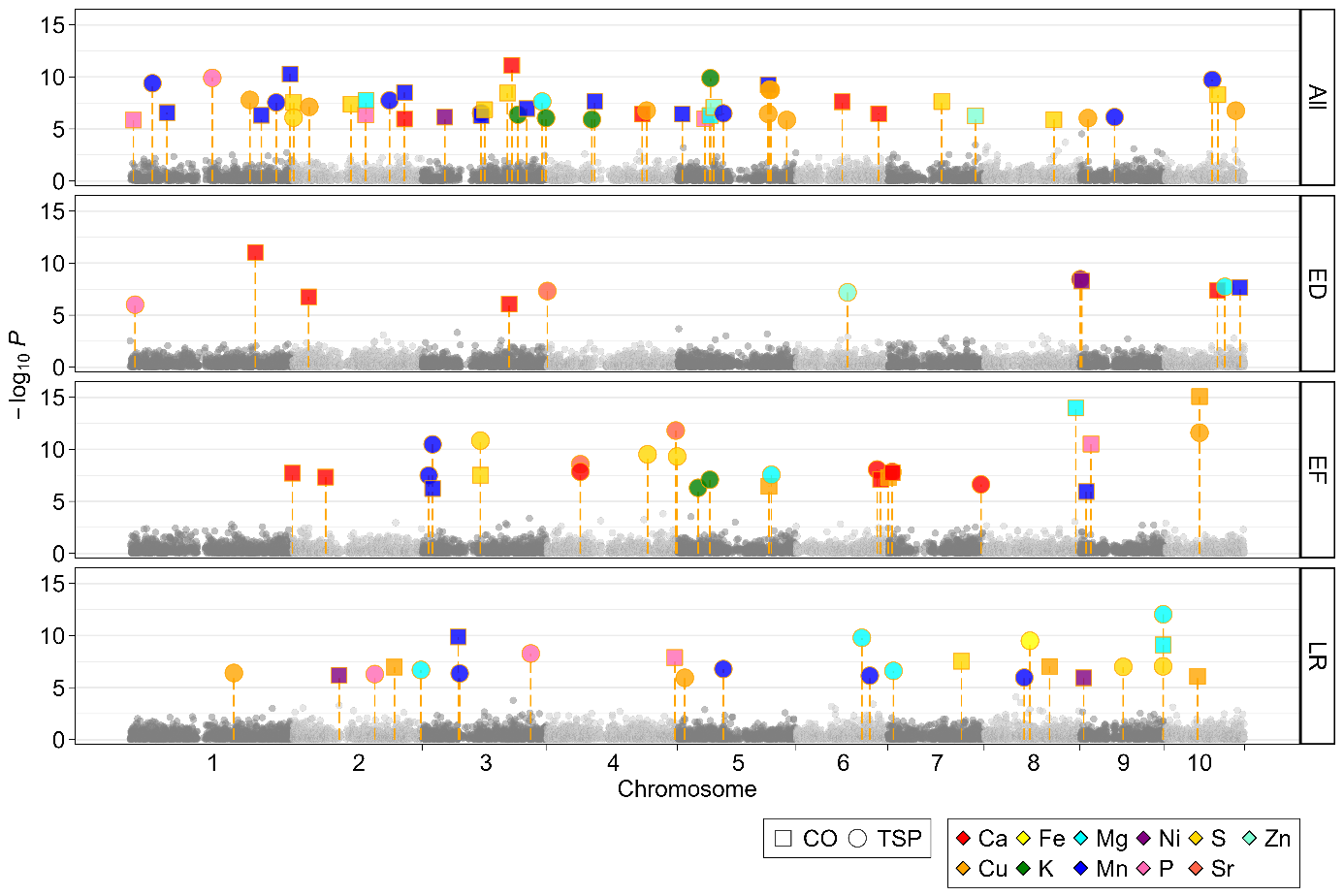


**Supp Figure 13 *Manhattan plot showing -log10(p-value) of SNP associations of mineral concentration traits in early biomass.*** *Highlighted dots indicate significant SNPs distinguished by their trait-specific color and treatment shape. All significant SNPs are plotted, while for non-significant SNPs every 100th position is shown.*


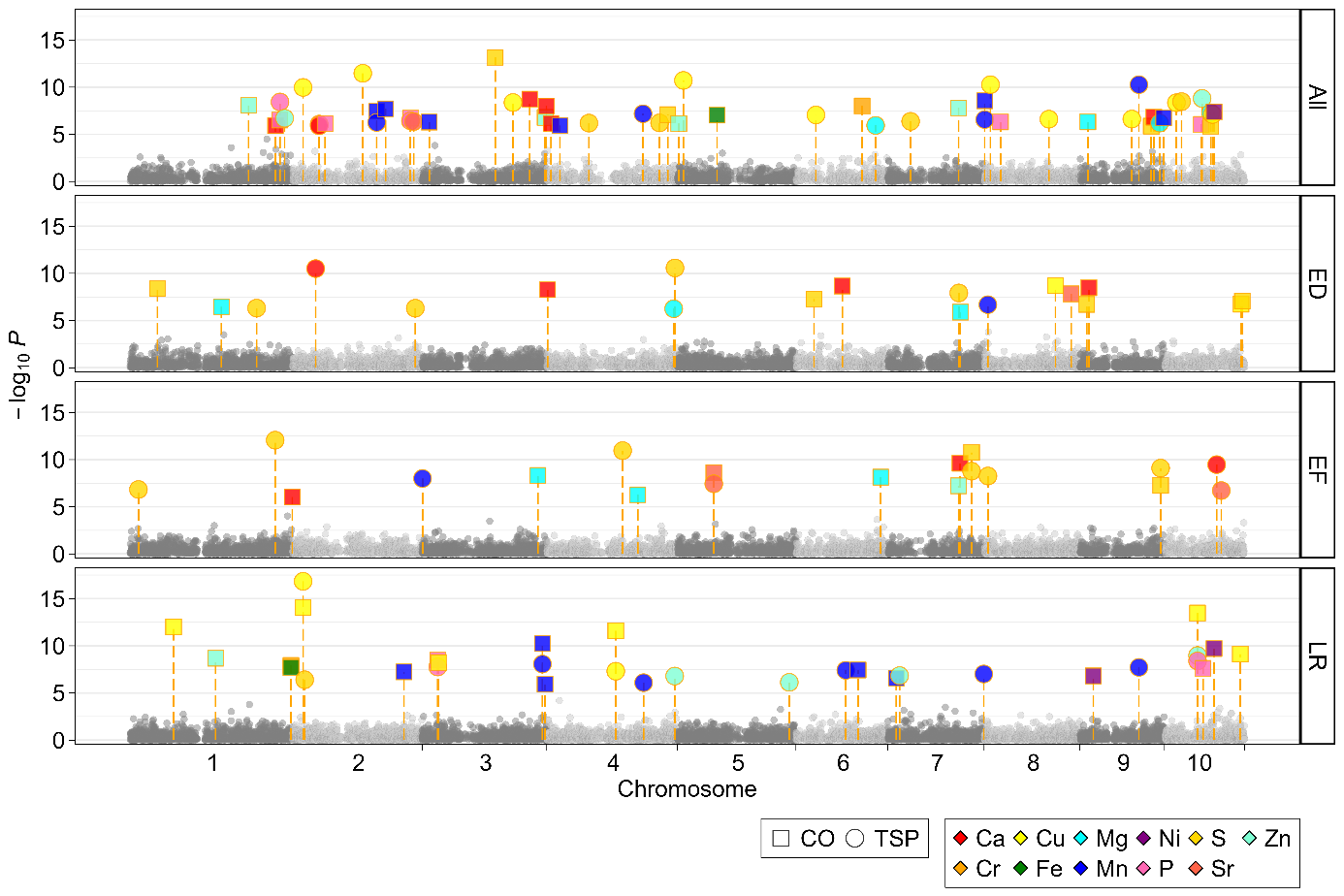


**Supp Figure 14 *Manhattan plot showing -log10(p-value) of SNP associations of mineral concentration traits in late biomass.*** *Highlighted dots indicate significant SNPs distinguished by their trait-specific color and treatment shape. All significant SNPs are plotted, while for non-significant SNPs every 100th position is shown.*


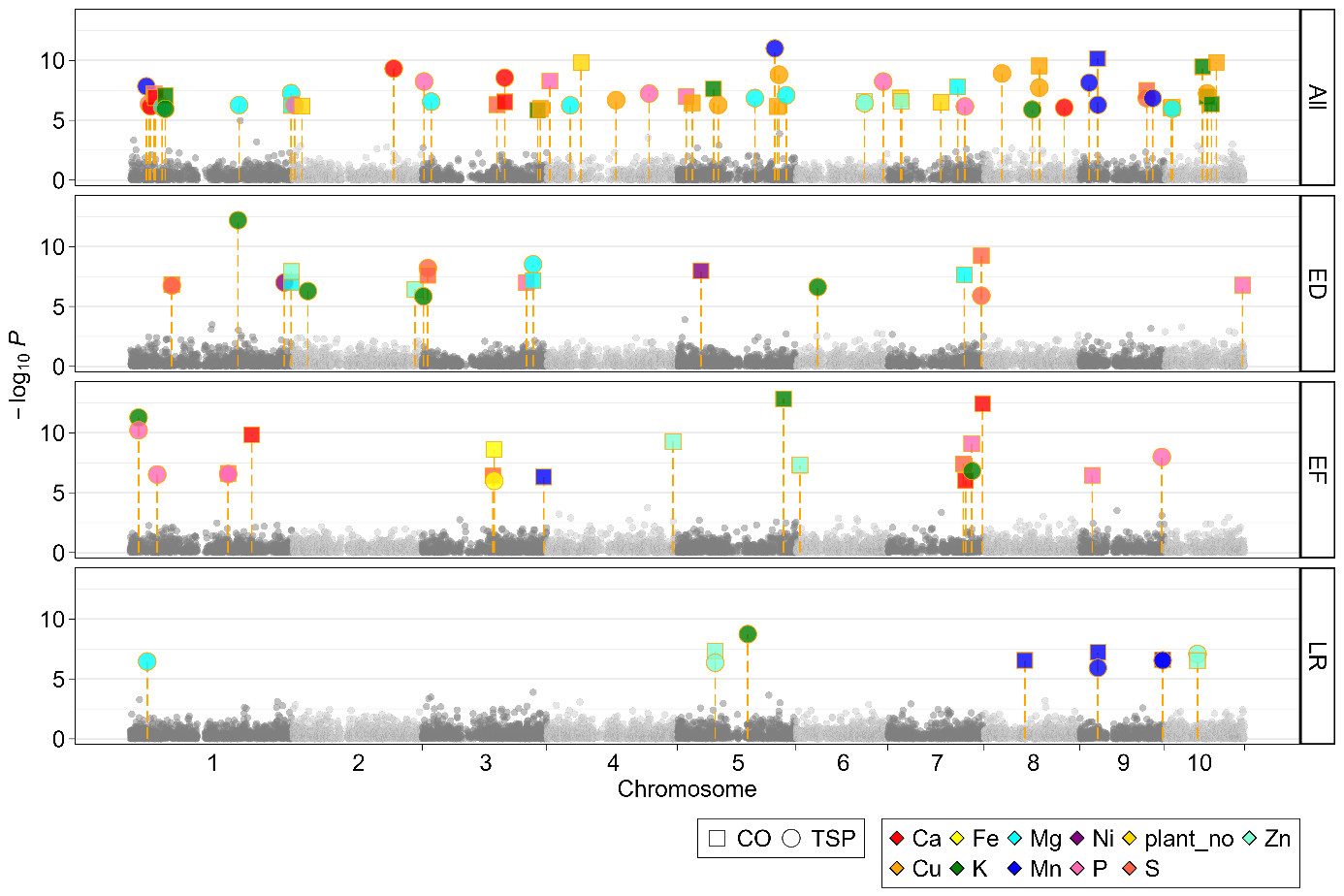


**Supp Figure 15 *Manhattan plot showing -log10(p-value) of SNP associations of mineral concentration traits in the grain.*** *Highlighted dots indicate significant SNPs distinguished by their trait-specific color and treatment shape. All significant SNPs are plotted, while for non-significant SNPs every 100th position is shown.*
